# Effects of Mechanical Dyssynchrony on Coronary Flow: Insights From a Computational Model of Coupled Coronary Perfusion With Systemic Circulation

**DOI:** 10.3389/fphys.2020.00915

**Published:** 2020-08-14

**Authors:** Lei Fan, Ravi Namani, Jenny S. Choy, Ghassan S. Kassab, Lik Chuan Lee

**Affiliations:** ^1^Department of Mechanical Engineering, Michigan State University, East Lansing, MI, United States; ^2^California Medical Innovation Institute, San Diego, CA, United States

**Keywords:** systemic circulation, coronary perfusion, mechanical dyssynchrony, ischemia, bi-directional interactions

## Abstract

Mechanical dyssynchrony affects left ventricular (LV) mechanics and coronary perfusion. Due to the confounding effects of their bi-directional interactions, the mechanisms behind these changes are difficult to isolate from experimental and clinical studies alone. Here, we develop and calibrate a closed-loop computational model that couples the systemic circulation, LV mechanics, and coronary perfusion. The model is applied to simulate the impact of mechanical dyssynchrony on coronary flow in the left anterior descending artery (LAD) and left circumflex artery (LCX) territories caused by regional alterations in perfusion pressure and intramyocardial pressure (*IMP*). We also investigate the effects of regional coronary flow alterations on regional LV contractility in mechanical dyssynchrony based on prescribed contractility-flow relationships without considering autoregulation. The model predicts that LCX and LAD flows are reduced by 7.2%, and increased by 17.1%, respectively, in mechanical dyssynchrony with a systolic dyssynchrony index of 10% when the LAD's *IMP* is synchronous with the arterial pressure. The LAD flow is reduced by 11.6% only when its *IMP* is delayed with respect to the arterial pressure by 0.07 s. When contractility is sensitive to coronary flow, mechanical dyssynchrony can affect global LV mechanics, *IMP*s and contractility that in turn, further affect the coronary flow in a feedback loop that results in a substantial reduction of *dP*_*LV*_/*dt*, indicative of ischemia. Taken together, these findings imply that regional *IMP*s play a significant role in affecting regional coronary flows in mechanical dyssynchrony and the changes in regional coronary flow may produce ischemia when contractility is sensitive to the changes in coronary flow.

## Introduction

Heart failure (HF) is a complex syndrome associated with significant morbidity, mortality and socioeconomic burden. Many HF patients are presented with mechanical dyssynchrony (Claridge et al., 2015) that is a strong predictor of cardiovascular mortality (Saporito and Fisher, 1987; Modin et al., 2018). Cardiac resynchronization therapy (CRT), on the other hand, has emerged as a powerful treatment for mechanical dyssynchrony and HF (Moss et al., 2009). About 30% of patients, however, still do not improve after therapy (non-responders) and the percentage of CRT non-responders has remained constant over the past decade (Gorcsan, 2011). The relatively high-rate of CRT non-responders has imposed a significant burden on the clinical course and outcome of patients (Corbisiero et al., 2016).

Studies have suggested that CRT response is sensitive to coronary perfusion (Trimble et al., 2008; Svendsen et al., 2012) and mechanical dyssynchrony elicits changes in coronary perfusion (Claridge et al., 2015). During normal electrical activation of the left ventricle (LV) in which contraction of the interventricular septum and LV free wall (LVFW) are synchronized, there is sufficient blood flow to balance the regional myocardial demand and supply (Borlaug and Kass, 2008). In left bundle branch block (LBBB), however, there is a delay in the electrical activation of the LVFW with respect to the septum that can produce acute changes in the LV function and coronary blood flow to the left anterior descending artery (LAD) (septal hypoperfusion) and left circumflex artery (LCX) territories in the LV (Fang et al., 2013; Claridge et al., 2015). Right ventricular (RV) pacing can also elicit mechanical dyssynchrony in which the septum and LVFW contract asynchronously (Heyndrickx et al., 1985), which also produces several abnormalities resembling that of LBBB [e.g., delayed LV relaxation, reduced cardiac output (Zile et al., 1987; Xiao et al., 1992), and depressed LV function (Shefer et al., 1987; Kolettis et al., 2000)]. Similar to LBBB, RV pacing can produce perfusion defects in the form of a reduction in LAD flow. This reduction has been found in patients without coronary artery disease (Matsumura et al., 1987) as well as in an animal model of RV pacing (Bailer, 1981). One study in LBBB patients with normal coronary arteries had an abnormal increase in lactate production during a high-rate of pacing, suggesting that perfusion changes associated with mechanical dyssynchrony may induce ischemia (Breithardt and Breithardt, 2012). It is, however, unclear if this ischemia, plausibly induced by changes in perfusion during mechanical dyssynchrony can reduce myocardial contractility, which in turn, causes further changes to the coronary flow that may exacerbate ischemia. Due to the confounding effects of the two-way interactions between LV mechanics and coronary flow during mechanical dyssynchrony, it is difficult to isolate the mechanisms behind these changes from experimental and clinical measurements alone.

To overcome the limitations associated with experiments and clinical studies of mechanical dyssynchrony (e.g., difficulty in decoupling the confounding effects between LV mechanics and coronary flow), computational models have been developed. Specifically, finite element (FE) models and lumped parameter models [e.g., TriSeg/CircAdap model (Arts et al., 2005)] of ventricular mechanics (Trayanova et al., 2011; Kerckhoffs et al., 2012; Sermesant et al., 2012; Walmsley et al., 2015; Lee et al., 2018; Arumugam et al., 2019) have been developed and applied to investigate global and regional LV mechanics (i.e., stresses and strains) and energetics (i.e., work done) in mechanical dyssynchrony. The impact of mechanical dyssynchrony on coronary flow, however, has not been incorporated, and correspondingly, interrogated in these models. This is in part because of the complexity and the need for a large number of parameters necessary to model the coupling between coronary perfusion and ventricular mechanics in a theoretical framework (Beard and Bassingthwaighte, 2000; Smith et al., 2002; Huo et al., 2009; Namani et al., 2018a). Moreover, most computational models of coronary perfusion (Beard and Bassingthwaighte, 2000; Smith et al., 2002; Huo et al., 2009; Namani et al., 2018a) are not coupled in a closed-loop manner with the systemic circulation and the heart. As a result, it is difficult to apply these models to directly investigate the bi-directional interaction mechanisms affecting the LV mechanics-coronary perfusion relationship, which is not completely understood in both normal and pathophysiological settings. This is especially significant since changes in the LV mechanics can affect coronary perfusion (via intramyocardial and perfusion pressures) and changes in coronary flow can affect LV mechanics (via changes in myocardial contractility) in a closed-loop feedback manner. Computational models that account for bi-directional interactions between the LV and the coronary network can, therefore, help discriminate the roles and contributions of the various confounding factors affecting coronary flow in mechanical dyssynchrony.

Motivated by the limitations of current models and the need to better understand the impact of mechanical dyssynchrony on coronary flow, we developed a novel computational framework that couples the systemic circulation and the LV's contraction-relaxation mechanics with the coronary flow dynamics in both the LAD and LCX territories. The framework is calibrated using experimental measurements of the coronary flow waveforms in the LAD and the LCX, pressure waveforms in the LV, as well as the LV volume waveform from a swine model under normal conditions. The calibrated model is then extended to model mechanical dyssynchrony with and without considering the effects of regional coronary flow on regional LV contractility (ischemia). A sensitivity analysis is performed by varying key parameters in the modeling framework [such as time delays in the regional intramyocardial pressure (*IMP*) waveforms] to investigate how they affect changes in the LAD and the LCX flows, as well as the global LV function in mechanical dyssynchrony. In addition, we investigate the role of the regional contractility—coronary flow relationship in producing regional ischemia that affects the (global and regional) LV mechanics, *IMP*, and contractility in mechanical dyssynchrony.

## Methods

### Experiment

All experiments were performed following the national and local ethical guidelines, including the Institute of Laboratory Animal Research guidelines, the Public Health Service policy, and the Animal Welfare Act, and an approved California Medical Innovations Institute IACUC protocol regarding the use of animals in research. Yorkshire domestic swine (*n* = 3), of either sex, were fasted overnight and sedated with TKX (Telazol 10 mg/kg, Ketamine 5 mg/kg, Xylazine 5 mg/kg; IM). The surgical plane was maintained with 1–2% Isoflurane and 100% O_2_ to keep PCO_2_ at 35–40 mmHg. Limb and precordial ECG leads were attached to the animals and cardiac electrical activity was monitored on a Physio-Control Lifepak-12 defibrillator. Two introducer sheaths were percutaneously inserted into the jugular vein for administration of fluids and placement of a pacing lead, and to access to the right side of the heart. An additional introducer sheath was percutaneously inserted into the right femoral artery to access the coronary arteries and the LV. Heparin 100 IU/kg was administered with IV fluids before further instrumentation of the animal, which was then supplemented as needed. Measurements were recorded with right atrial (RA) pacing at 100 beats per minute.

#### Left Ventricular Pressure

After proper calibration, a Millar pressure catheter (Ventri Cath 507), which was connected to an MPVS Ultra PV loop system (Millar, Houston, Texas, USA), was advanced into the LV using a guide catheter. Pressure waveforms were recorded using a data acquisition system (LabChart Pro, ADInstruments, Colorado Springs, CO, USA).

#### Echocardiography

Transesophageal echocardiograms were obtained simultaneously at the time of LV pressure measurements, using an EPIQ 7C ultrasound system (Philips, Andover, MA) with an X8-2t transducer. Two- and three-dimensional echocardiographic images in the long axis view were acquired with the animal placed in the supine position. The images were analyzed offline using QLAB 10.8 (Philips, Andover, MA) to determine ejection fraction (EF), LV volume waveform, cardiac output, and stroke volume.

#### Coronary Flow Rate

The chest was opened through a midline sternotomy and an incision was made in the pericardium with the creation of a sling to support the heart. The LAD and LCX arteries were carefully dissected free from their surrounding tissue and a flow probe connected to a flow meter (Transonic, Ithaca, NY, USA) was placed around the arteries to measure their flow rates during RA pacing.

### Cardiac-Coronary Modeling Framework

**Figure 1:**
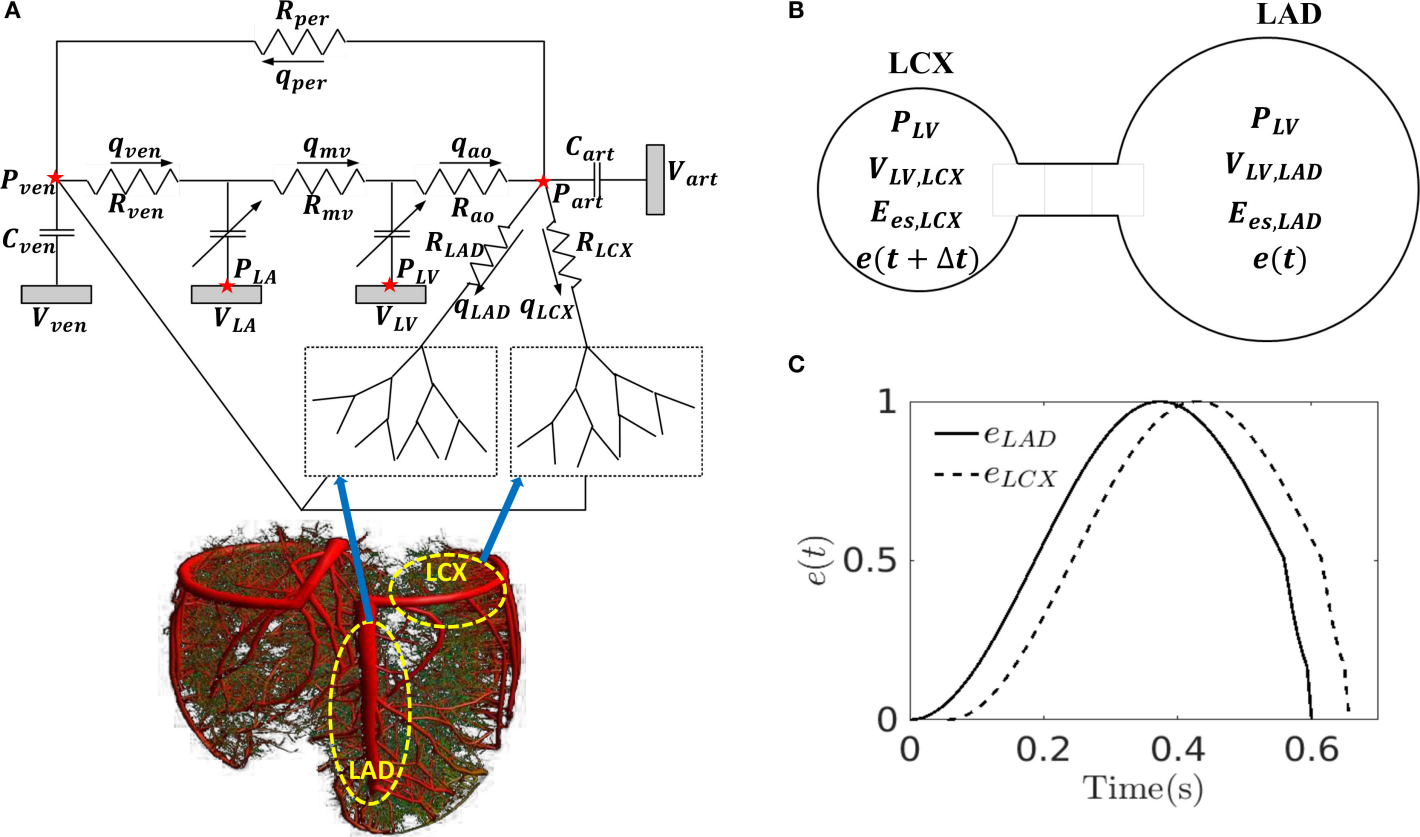
**(A)** Schematic of the cardiac-coronary modeling framework: the LV and the coronary tree (LAD and LCX) are connected to a closed-loop lumped model with their pressures (*P*), volumes (*V*), flow rate (Q), compliances (*C*), and resistances (*R*) where ao, art, ven, and mv denote the aorta, peripheral arteries, peripheral veins, and mitral valves, respectively; **(B)** A representation of the model to separate the LV into LAD and LCX compartments with their pressures (*P*), volumes (*V)*, maximal chamber elastances (*E*_*es*_), and time-varying elastances (*e*); **(C)** Time-varying elastance function in the LAD and LCX, *e*_*LAD*_ and *e*_*LCX*_ in mechanical dyssynchrony. Note, the LAD and LCX networks are graphically depicted by the 3D coronary tree in the lower left figure.

The closed-loop lumped parameter modeling framework consists of the LV, systemic and coronary circulations to describe the bi-directional interactions between LV mechanics and coronary perfusion. A schematic of the modeling framework is given in Figure 1A, showing the connection between the LV and systemic circulations with coronary network.

#### Systemic Circulation

The systemic circulation consists of four key compartments: LV, left atrium (LA), peripheral arterial and venous networks that are each represented by their corresponding electrical analogs (Figure 1A) (Shavik et al., 2017, 2018). Total blood volume is conserved by relating the rate of volume change in each storage compartment in the circulatory system to the inflow and outflow rates as

(1) $$\begin{array}{l} \frac{dV_{LA}\left(t\right)}{dt}=q_{ven}\left(t\right)-q_{mv}\left(t\right), \end{array}$$

(2) $$\begin{array}{l} \frac{dV_{LV}\left(t\right)}{dt}=q_{mv}\left(t\right)-q_{ao}\left(t\right), \end{array}$$

(3) $$\begin{array}{l} \frac{dV_{art}\left(t\right)}{dt}=q_{ao}\left(t\right)-q_{per}\left(t\right)-q_{LAD}\left(t\right)-q_{LCX}\left(t\right), \end{array}$$

(4) $$\begin{array}{l} \frac{dV_{ven}\left(t\right)}{dt}=q_{per}\left(t\right)-q_{ven}\left(t\right){+\;q}_{LAD}\left(t\right)+q_{LCX}\left(t\right). \end{array}$$

In Equations (1)–(4), *V*_*LA*_, *V*_*LV*_, *V*_*art*_, and *V*_*ven*_ are the volumes of the four compartments with the subscripts denoting the LA, LV, peripheral arteries, and peripheral veins, respectively. In Equations (3) and (4), *q*_*LAD*_ and *q*_*LCX*_ are the coronary flow rates associated with the LAD and LCX territories, respectively. Flow rates in the venous, mitral valve, aortic, and peripheral arteries are denoted by *q*_*ven*_, *q*_*mv*_, *q*_*ao*_, and *q*_*per*_, which are each determined by their segment's resistance and the pressure difference between the two connecting storage compartments as

(5) $$\begin{array}{l} q_{ao}\left(t\right)=\{\begin{array}{c} \frac{P_{LV}\left(t\right)-P_{art}\left(t\right)}{R_{ao}}\;P_{LV}\left(t\right)\geq P_{art}\left(t\right)\\ 0\;P_{LV}\left(t\right)<P_{art}\left(t\right) \end{array}, \end{array}$$

(6) $$\begin{array}{l} q_{per}\left(t\right)=\frac{P_{art}\left(t\right)-P_{ven}\left(t\right)}{R_{per}}, \end{array}$$

(7) $$\begin{array}{l} q_{ven}\left(t\right)=\frac{P_{ven}\left(t\right)-P_{LA}\left(t\right)}{R_{ven}}, \end{array}$$

(8) $$\begin{array}{l} q_{mv}\left(t\right)=\{\begin{array}{c} \frac{P_{LA}\left(t\right)-P_{LV}\left(t\right)}{R_{mv}}\;P_{LA}\left(t\right)\geq P_{LV}\left(t\right)\\ 0\;P_{LA}\left(t\right)<P_{LV}\left(t\right) \end{array}, \end{array}$$

where *P*_*LV*_, *P*_*art*_, *P*_*ven*_, and *P*_*LA*_ are the cavity pressures, and *R*_*ao*_, *R*_*per*_, *R*_*ven*_, and *R*_*mv*_ are the resistances of the aorta, peripheral arteries, peripheral veins, and mitral valves, respectively. We note that the flows across the heart valves are unidirectional (assuming no valvular regurgitation) (Shavik et al., 2019). Pressures in the storage compartments representing the peripheral arterial and venous networks are given as

(9) $$\begin{array}{l} P_{ven}\left(V_{ven}\left(t\right),\;t\right)=\frac{V_{ven}\left(t\right)-V_{ven0}}{C_{ven}}, \end{array}$$

(10) $$\begin{array}{l} P_{art}\left(V_{art}\left(t\right),\;t\right)=\frac{V_{art}\left(t\right)-V_{art0}}{C_{art}}, \end{array}$$

where (*V*_*art*0_, *V*_*ven*0_) are the prescribed (arteries and veins) resting volumes, and (*C*_*art*_, *C*_*ven*_) are the prescribed total (arteries and veins) compliances. The pumping characteristics of the LV and LA are represented by a time-varying elastance model (Santamore and Burkhoff, 1991; Witzenburg and Holmes, 2018). For the LA, instantaneous pressure, *P*_*LA*_ is related to the instantaneous volume, *V*_*LA*_ by a time-varying elastance function, *e*_*LA*_(*t*) as follows

(11) $$\begin{array}{l} P_{LA}\left(V_{LA}\left(t\right),\;t\right)=e_{LA}\left(t\right)P_{es}\left(V_{LA}\left(t\right)\right)\\ \;+\left(1-e_{LA}\left(t\right)\right)P_{ed}\left(V_{LA}\left(t\right)\right), \end{array}$$

where

(12) $$\begin{array}{l} P_{es}\left(V_{LA}\left(t\right)\right)=E_{es,LA}\left(V_{LA}\left(t\right)-V_{LA0}\right), \end{array}$$

(13) $$\begin{array}{l} P_{ed}\left(V_{LA}\left(t\right)\right)=A_{LA}\left\{\exp\left[B_{LA}\left(V_{LA}\left(t\right)-V_{LA0}\right)\right]-1\right\}, \end{array}$$

(14) $$\begin{array}{l} e_{LA}\left(t\right)=\{\begin{array}{ll} \frac{1}{2}\left[\left(\sin\frac{\pi }{T_{max,LA}}t-\frac{\pi }{2}\right)+1\right] & t<\frac{3}{2}T_{max,LA}\\ \frac{1}{2}\exp\left[-\left(t-\frac{3T_{max,LA}}{2}\right)\frac{1}{\tau }\right] & t\geq \frac{3}{2}T_{max,LA} \end{array}. \end{array}$$

In Equation (11), *P*_*es*_ and *P*_*ed*_ are the end-systolic and end-diastolic pressures, respectively, *E*_*es,LA*_ is the maximal chamber elastance of the LA, *V*_*LA*0_ is the volume with zero end-systolic pressure, and both *A*_*LA*_ and *B*_*LA*_ are parameters defining the end-diastolic pressure volume relationship of the LA. In Equation (14), *T*_*max,LA*_ is the time to end systole and τ_*LA*_ is the relaxation time constant.

In order to simulate asynchronous activation of the septum associated with the LAD territory and the LVFW associated with the LCX territory, we divided the LV cavity volume (*V*_*LV*_) into two compartments with volumes (*V*_*LV,LAD*_) and (*V*_*LV,LCX*_) based on the approach by Sunagawa et al. (1983) (i.e., *V*_*LV*_ = *V*_*LV, LAD*_ + *V*_*LV,LCX*_) (Figure 1B) and assumed the pressures in the compartments associated with the LAD and LCX to be equal to the LV cavity pressure (*P*_*LV*_). In Sunagawa's study on ischemia (Sunagawa et al., 1983), LV pressure was derived by prescribing different *E*_*es*_ in the normal and ischemic regions. Here, based on the same assumption (i.e., *P*_*LV*_ = *P*_*LAD*_ = *P*_*LCX*_), different time-varying elastance functions (*e*_*LAD*_, *e*_*LCX*_) and different maximal chamber elastances (*E*_*es,LAD*_, *E*_*es,LCX*_) are prescribed for the LAD and LCX territories. To simulate mechanical dyssynchrony, we prescribed a positive time delay (Δ*t* > 0) in the time-varying elastance function of the LCX with respect to the LAD [i.e., *e*_*LCX*_ = *e*(*t* + Δ*t*) and *e*_*LAD*_ = *e*(*t*)] so that the contraction of LCX territory happens later than that in the LAD territory with respect to end of diastole (Figure 1C). The resultant LV cavity pressure is given as

(15) $$\begin{array}{l} P_{LV}=[\left(V_{LV}-V_{LV0}\right)-\frac{F\left(1-e\left(t\right)\right)P_{ed}}{E_{es,LAD}\;e\left(t\right)}\\ \;-\frac{\left(1-F\right)\left(1-e\left(t+\Delta t\right)\right)P_{ed}}{E_{es,LCX}\;e\left(t+\Delta t\right)}]\\ \;\frac{E_{es,LAD}\;e\left(t\right)E_{es,LCX}\;e\left(t+\Delta t\right)}{{FE}_{es,LCX}\;e\left(t+\Delta t\right){+\left(1-F\right)E}_{es,LAD}\;e\left(t\right)}, \end{array}$$

where *F* = *V*_*LV,LAD*_/*V*_*LV*_ is a prescribed value, and *V*_*LV*0_ is the LV volume at zero end-systolic pressure (refer to Appendix A for derivation). We note that the zero end-systolic pressure corresponds to the volume intercept of the end-systolic pressure volume relationship (ESPVR), which we have assumed to be linear (Davidson et al., 2015). In Equation (15), *P*_*es*_, *P*_*ed*_, *e*(*t*), and *e*(*t* + Δ*t*) are obtained from Equations (12)–(14) by replacing the subscript *LA* with *LV*.

#### Coronary Circulation

**Figure 2:**
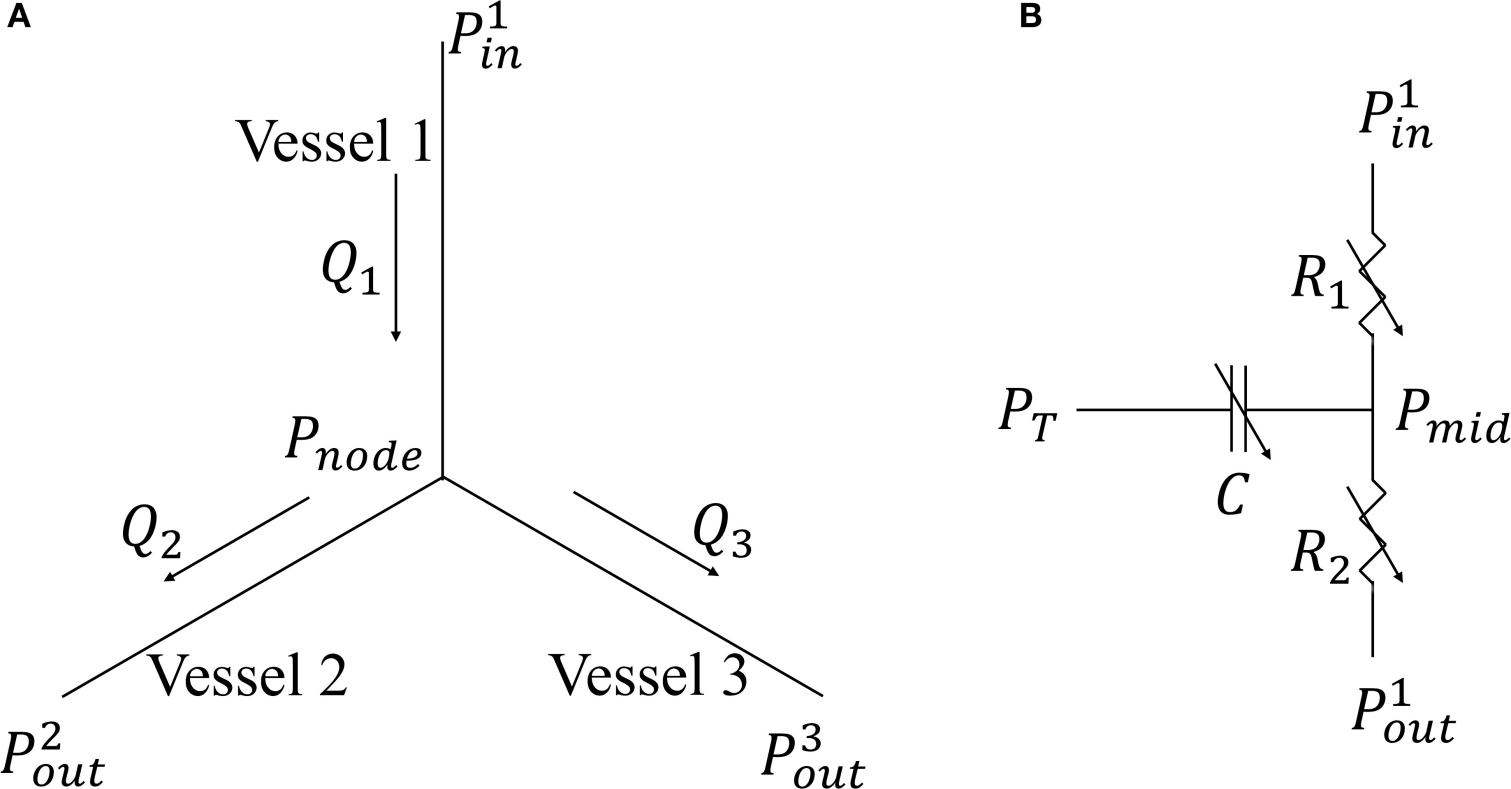
**(A)** Single bifurcation three-vessel network; **(B)** Electrical non-linear analog of a single vessel.

Coronary network morphometry of the LAD and LCX territories are each described using a previous coronary arterial model (Namani et al., 2018a). Morphometry of the arterial tree in that model is pruned from a previously reconstructed coronary arterial network and consists of 400 vessels (from order 6 to order 0) with 195 bifurcations, 3 trifurcations, and 79 terminal vessels (Kaimovitz et al., 2010). The microvascular tree, an input to the flow analysis, was generated from the global statistics of the measured coronary vessel diameters (Kassab et al., 1993; Kaimovitz et al., 2005, 2010). Because there are many capillary networks at the distal end of the coronary arterial tree, the terminal vessels of the reconstructed tree are truncated with either pre-capillary arterioles of vessel order 1 and/or capillary arterioles of vessel order 0. Larger arterioles are designated as vessels of orders 1, 2, 3, …, 6 in the direction of increasing diameter (Kassab et al., 1993). To maintain physiological flow dispersion levels, the diameter of each vessel order was reassigned within the range of the measurement in the reconstructed microvascular tree as described previously (Namani et al., 2018b). To take into account the differences in the morphometry (e.g., diameter and length of the vessels) and flow in the LAD vs. the LCX, we prescribed different proximal resistances *R*_*LAD*_ and *R*_*LCX*_ in the corresponding segments connecting the coronary networks to the systemic circulation (Figure 1A) (Kassab et al., 1993). To couple the coronary perfusion (both the LAD and the LCX networks) with the systemic circulation, the arterial pressure (*P*_*art*_) and venous pressure (*P*_*ven*_) are employed as the inlet and outlet pressure boundary conditions of the coronary arterial tree, based on the fact that (1) most of the pressure drop occurs across the coronary arterial microvascular networks (Marcus et al., 1990) and (2) the systemic venous pressure (~10 mm Hg) is of the same magnitude as the RA pressure that the networks drain into under physiological conditions. We also note that the venous pressure is imposed at the outlet pressure in the “intramyocardial pump” model introduced by Spaan et al. (1981). The model is solved for coronary flows in the LAD (*q*_*LAD*_) and the LCX (*q*_*LCX*_) trees, which are fed into the systemic circulation model in Equations (3) and (4). In the flow analysis, it is assumed that the diameter is homogeneous within each vessel segment but different from vessel to vessel. The non-linear three-element Windkessel electrical representation that has been investigated previously (Namani et al., 2018a) is employed in this work for coronary network analysis. Modeling the flow in a trifurcation is analogous to that in a bifurcation (Namani et al., 2018a), with the difference being that an additional vessel needs to be accounted for in the mass conservation. For a single bifurcation (*j* = 3) or trifurcation network (*j* = 4), mass conservation is satisfied at the nodal position as

(16) $$\begin{array}{l} \overset{j}{\underset{i=1}{\sum }}Q_{i}=0, \end{array}$$

where *Q*_*i*_ is the flow in each vessel (Figure 2A). For each single vessel *i*, using mass conservation at Windkessel's internal (“mid”) node, the ordinary differential equation in terms of the flow is

(17) $$\begin{array}{l} \sum Q_{mid}=\frac{P_{in}^{i}-P_{mid}^{i}}{R_{1}^{i}}+\frac{P_{out}^{i}-P_{mid}^{i}}{R_{2}^{i}}\\ \;+\frac{d}{dt}\left[C^{i}\left(P_{T}^{i}-P_{mid}^{i}\right)\right]=0, \end{array}$$

where *P*_*mid*_ is the unknown pressure in the geometric center of the vessel, *P*_*T*_ is the intramyocardial pressure with details found in the next section, the resistances *R*_1_ and *R*_2_ (assumed to be the same) and the capacitor *C* are obtained by

(18) $$\begin{array}{l} R_{1}=R_{2}=\frac{64\mu \;L}{\pi \cdot {D\left(t\right)}^{4}} \end{array}$$

and

(19) $$\begin{array}{l} C=\frac{\partial V}{\partial \left(P_{mid}\left(t\right)-P_{T}\left(t\right)\right)}=\frac{\partial \left(\pi \cdot {D\left(t\right)}^{2}L/4\right)}{\partial \left(P_{mid}\left(t\right)-P_{T}\left(t\right)\right)}. \end{array}$$

In Equation (18), *L* is the length and *D*(*t*) is the diameter of single coronary vessel, respectively, and μ denotes the effective blood viscosity, which is assumed to be a constant to simplify the flow analysis (Pries et al., 1994) (Figure 2B). The capacitance of an intramyocardial arteriole is non-linear and defined by Equation (19) where the partial derivative means the change in lumen volume (V) with respect to the transvascular pressure when the other variables affecting the lumen volume are not varied (Jacobs et al., 2008). The vessel diameter is a sigmoidal function of transvascular pressure as

(20) $$\begin{array}{l} D\left(t\right)=2\left\{B_{p}+\frac{A_{p}-B_{p}}{\pi }\left[\frac{\pi }{2}+arctan\left(\frac{\Delta P-\varphi_{p}}{C_{p}}\right)\right]\right\}, \end{array}$$

where *A*_*p*_ and *B*_*p*_ are the asymptotical highest and lowest radii, respectively (under the highest and lowest transvascular pressure), φ_*p*_ is the transvascular pressure corresponding to the mean of radii *A*_*p*_ and *B*_*p*_, and *C*_*p*_ is the passive response bandwidth. For the parameters used in Equation (20), refer to Namani et al. (2018a). As the resistance and capacitor in the network is not a constant and depend on the flow conditions, the resulting system of ordinary differential equations is non-linear.

#### Myocardium Vessel Interaction

In the model, intramyocardial pressure $P_{T}^{i}$ in each vessel (i.e., extravascular forces on the coronary vessels) of the LAD and LCX arterial trees are prescribed to be homogeneous and have the value of *IMP*_*LAD*_ and *IMP*_*LCX*_, respectively, that are defined as follows. Because the distribution of *IMP in vivo* is difficult to measure (Heineman and Grayson, 1985; Mihailescu and Abel, 1994), some studies have assumed that the *IMP* equals the LV pressure at the endocardium and decreases linearly to zero at the epicardium (Downey and Kirk, 1975; Bellamy, 1982). On the other hand, it has been suggested that the LV pressure is not the sole determinant of *IMP* but myocardial contraction (associated with changing myocardial elasticity and myocyte shortening) also affects the coronary flow myocardium-vessel interaction mechanisms (Westerhof, 1990; Westerhof et al., 2006): (1) time-varying elasticity (VE), (2) myocardial shortening-induced intracellular pressure (SIP), and (3) ventricular cavity-induced extracellular pressure (CEP) (Algranati et al., 2010). Correspondingly, the intramyocardial pressure is prescribed as

(21) $$\begin{array}{l} IMP_{\left(LAD,LCX\right)}=\underset{\text{CEP}}{\underset{︸}{\alpha P_{LV}}}+\underset{\text{VE}}{\underset{︸}{\beta E_{es,\left(LAD,LCX\right)}e_{\left(LCX,LAD\right)}}}\\ \;+\underset{\text{SIP}}{\underset{︸}{\gamma \left(1-SSR\right),}} \end{array}$$

where α, β, and γ are prescribed parameters, and *SSR* is the myocardial stretch. These parameters are prescribed based on a sensitivity analysis (refer to Appendix E) to (1) best match the coronary flow rate waveform and (2) be consistent with experiments. Specifically, α is constrained between 0 and 1 based on previous modeling studies (Arts and Reneman, 1985; Bruinsma et al., 1988) that assume CEP to vary linearly from the cavity pressure *P*_*LV*_ at the endocardium to zero at the epicardium. The parameter γ is chosen to produce a peak SIP that is about 20% of *IMP* based on a previous study (Mynard et al., 2014). For the SIP component in Equation (21), *SSR* is defined as the ratio of *V*_*LV*_ to the LV's end-diastolic volume (Algranati et al., 2010). The sum of peak SIP and VE produced by any combination of α, β and γ should be <30% of *IMP* based on the experimental data by Rabbany et al. (1989), which shows that the peak *IMP* is 10–30% greater than the peak *P*_*LV*_. Based on these conditions and sensitivity analysis, we arrived at the values α = 0.8, β = 5, and γ = 20. Here, we note that the different time-varying elastance functions (*e*_*LAD*_, *e*_*LCX*_) prescribed to simulate mechanical dyssynchrony will result in different *IMP*s on the LAD and the LCX networks.

### Simulations for Mechanical Dyssynchrony and Ischemia

We consider three types of simulations: (1) control (CON) based on measurements from three RA pacing swine models, (2) isolated mechanical dyssynchrony (MD), and (3) mechanical dyssynchrony + ischemia (MD + IS). In the control simulations (without mechanical dyssynchrony) (*n* = 3), model parameters were calibrated to fit the coronary flow (LAD, LCX), LV volume and pressure, and arterial pressure waveforms measured in the animals. Specifically, the peak LV pressure and end-diastolic LV pressure were matched by manually adjusting the aorta and mitral valve resistances, (*R*_*ao*_ and *R*_*mv*_), and the passive parameters defining the end-diastolic pressure volume relationship (*A*_*LV*_ and *B*_*LV*_). The arterial compliance (*C*_*art*_), the peripheral arterial resistance (*R*_*per*_), the arterial resting volume (*V*_*art*0_), and the initial value for the arterial volume (*V*_*art*_) were manually adjusted to match both the peak and the end systolic arterial pressures. The LV volume waveform was matched by manually adjusting the resting LV volume (*V*_*LV*0_), the initial value of the LV volume, (*V*_*LV*_) and the venous vessel compliance (*V*_*ven*_). For the control simulations, *E*_*es,LAD*_ was set equal to *E*_*es,LCX*_ and Δ*t* was set to zero. Therefore, the LAD and LCX networks are exposed to the same *IMP*, and flows in the LAD and LCX were matched by altering their corresponding inlet resistances (*R*_*LAD*_ and *R*_*LCX*_). Based on the initial guess of the volume in each compartment (*V*_*ven*_, *V*_*art*_, *V*_*LV*_, *V*_*LA*_), the simulation is run until the LV pressure, volume, and coronary flow rate waveforms are at steady-state time-periodic conditions. Reference parameters used in previous studies on lumped-parameter models (Shavik et al., 2019; Namani et al., 2020) are selected and manually varied after each run until the waveforms are in agreement with the experimental data. We note that parameters for the coronary model (section Coronary Circulation) were obtained from a previous study and fixed here whereas parameters associated with IMP (α, β and γ) were obtained based on a sensitivity study as discussed earlier. Only parameters associated with the lumped parameter model of the systemic circulation and the proximal resistances (*R*_*LAD*_ *and R*_*LCX*_) of the coronary arterial tree are calibrated based on the experimental measurements. The parameters from the calibrated model for the three swine model are listed in Appendix B **(Table B1)**. We note that *V*_*LV*0_ (volume intercept of the prescribed linear ESPVR) is negative in **Table B1** because the time-varying elastance model does not take into account the non-linearity of ESPVR at small *V*_*LV*_. We further note that a negative *V*_*LV*0_ has been found experimentally and numerically (Crottogini et al., 1987; Pironet et al., 2013; Davidson et al., 2015). Parameters that are fixed for all simulations are listed with their value in Table 1.

**Table 1:** Parameters used in the time-varying elastance model.

**Parameter**	**Unit**	**Value**
*E*_*es,LAD*_; *E*_*es,LCX*_	mmHg/ml	3.18
*E*_*es,LA*_	mmHg/ml	1.50
*T*_*max,LV*_	s	2.00 × 10^−1^
*T*_*max,LA*_	s	1.25 × 10^−1^
*A*_*LV*_	mmHg	1.28 × 10
*B*_*LV*_	ml^−1^	1.50 × 10^−2^
*A*_*LA*_	mmHg	4.40 × 10^−2^
*B*_*LA*_	ml^−1^	4.90 × 10^−2^

The calibrated model is then applied to study the independent effect of mechanical dyssynchrony (MD) and its combined effect with ischemia (MD + IS) on LV function. Since there is a delayed activation in the LCX in the MD simulations (*n* = 3), we prescribed a systolic dyssynchrony index (SDI > 0), which is defined by the time delay Δ*t* (Δ*t* = duration of one cardiac cycle × SDI) to model the delayed contraction (see Equation 15) of the LCX with respect to the LAD territory. We note that SDI is a common metric used to assess mechanical dyssynchrony in the clinic (Delgado et al., 2008; Tani et al., 2012). Additionally, we also prescribed a different time delay $\Delta \bar{t}$ for both the elastance functions [i.e., $e_{LCX}=e\left(t+\Delta t+\Delta \bar{t}\;\right)$ and $e_{LAD}=e\left(t+\Delta \bar{t}\;\right)]$ in the IMP function in Equation (21) to simulate the effects of time delay on the contraction of the LAD territory with respect to *P*_*LV*_ (whose elastance function in Equation 15 is not altered).

**Figure 3:**
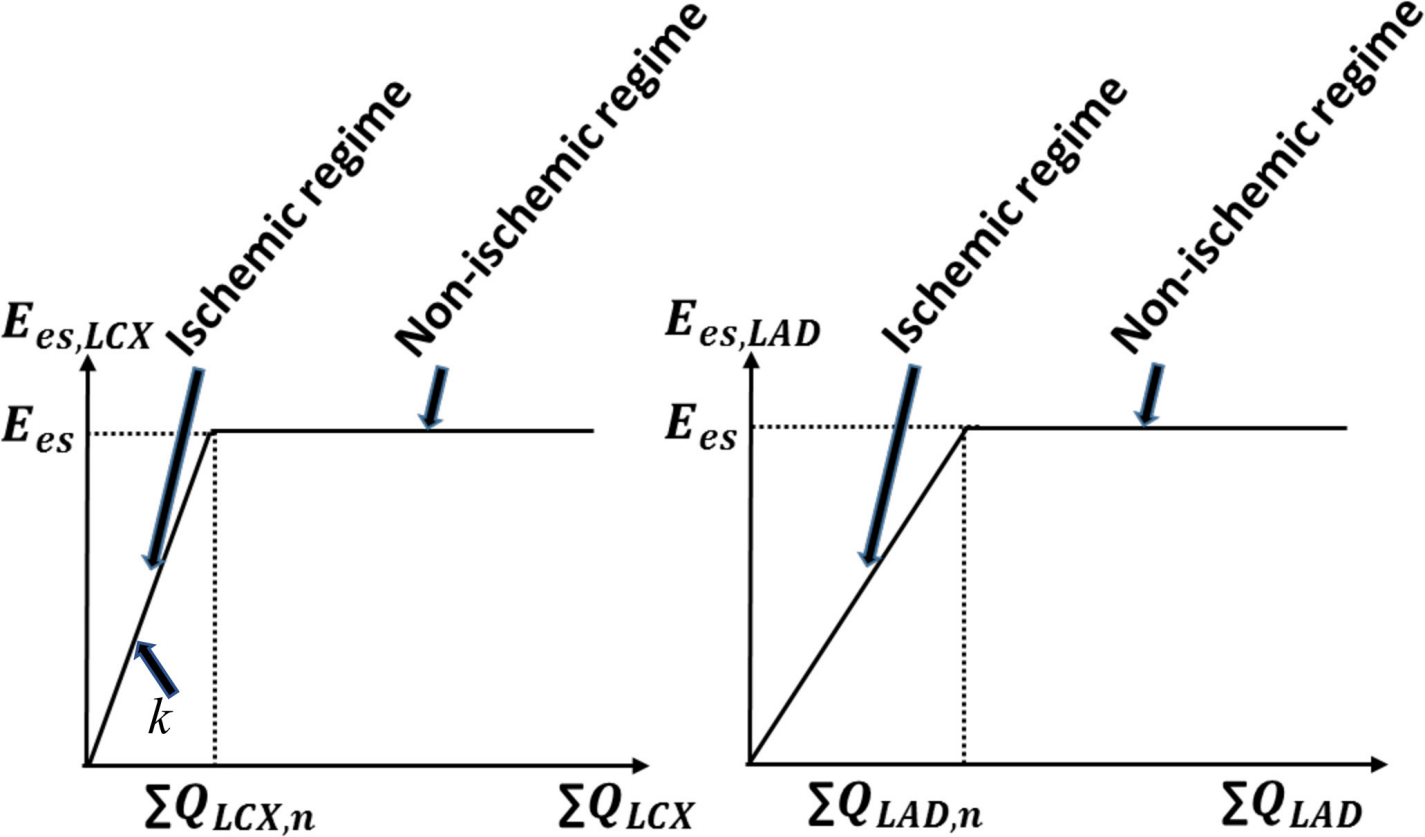
The prescribed contractility-coronary flow relationship in both the ischemic and non-ischemic myocardial regimes. The parameter “*k*” represents the slope of this relationship in the ischemic regime and “n” denotes normal condition.

In the MD + IS simulations (*n* = 3), we include the bi-directional interaction between the coronary flow and the LV contractility. To model this interaction, we prescribed a relationship between contractility and the coronary flow separately in the LAD and LCX territories based on experimental observations (Figure 3) (Gallagher et al., 1984; Ross, 1991; Heusch and Schulz, 1999). This relationship consists of two regimes, an ischemic and a non-ischemic one that are separated by the transitional flows ∑ *Q*_*LAD, n*_ and ∑ *Q*_*LCX, n*_ in the LAD and the LCX, respectively. The transitional flows ∑ *Q*_*LAD, n*_ and ∑ *Q*_*LCX, n*_ are prescribed, respectively, by the corresponding total coronary flow (over a cardiac cycle) measured in the normal swine model (“n” denotes to normal). In the ischemic regime, maximal chamber elastances of the LAD (*E*_*es,LAD*_) and LCX (*E*_*es,LCX*_) territories vary linearly with their corresponding total coronary flows (over a cardiac cycle) ∑ *Q*_*LAD*_ and ∑ *Q*_*LCX*_, respectively. The ischemic regime is controlled by the slope *k*, which has different values in the LAD and LCX (Figure 3). In the non-ischemic regime, the maximal chamber elastances remains constant with respect to their corresponding coronary flows (Heusch, 2019).

This linear relationship between contractility and coronary flow in the ischemic regime is constructed based on ample and robust experimental evidence of “perfusion-contraction matching” in the ischemic myocardium as described by Schulz et al. (1989) and Ross (1991). Considering the prescribed contractility-flow relationship, coronary flow alterations caused by mechanical dyssynchrony affect regional contractility (*E*_*es,LAD*_, *E*_*es,LCX*_) that further affects both *IMP*s in the LAD and the LCX networks, and the coronary networks' inlet pressures, which changes the coronary flow again in a closed-loop feedback. To elucidate the complex effects quantitatively, we consider two cases with different values of the contractility-flow gradient *k*.

We note that the parameters given in Table 1 and the animal specific parameters in **Table B1** are fixed for the three simulation cases. All simulations are run with a heart rate at 100 beats per minute for several cardiac cycles until the pressure-volume loop reaches a steady state.

## Results

### Experimental Measurements

Experimental measurements and model predicted results of the coronary flow in LAD (∑ *Q*_*LAD*_) and LCX (∑ *Q*_*LCX*_), LV end-diastolic volume (LV EDV), and peak LV pressure (peak LVP) from three swine are summarized in Table 2. The LV EDV and peak LVP are within the normal range as compared to a previous work (Lionetti et al., 2013). Coronary flow measured in the LAD is larger than that in the LCX in the three swine models. This is consistent with previous studies (Seiler et al., 1992; Miguel, 1999), where the epicardial vessel in the LAD network has been observed to have a larger diameter than that in the LCX network. This allows for more flow in the LAD artery as volumetric flow is proportional to the fourth power of the vessel's diameter.

**Table 2:** Experimental measurements and model predictions of three swine models.

**Swine**	**1**	**2**	**3**	**Mean ± SD**
**Experimental measurements**				
∑ *Q*_*LAD*_ (ml)	18.09	11.62	18.61	16.16 ± 3.86
∑ *Q*_*LCX*_ (ml)	9.38	8.27	14.54	10.65 ± 3.26
LV EDV (ml)	50.95	44.18	53.83	49.65 ± 4.95
Peak LVP (mmHg)	107.31	114.12	107.21	109.55 ± 3.96
**Model predictions**				
∑ *Q*_*LAD*_ (ml)	18.09	11.71	18.68	16.16 ± 3.86
∑ *Q*_*LCX*_ (ml)	9.48	8.13	14.33	10.65 ± 3.26
LV EDV (ml)	49.61	42.61	56.20	49.47 ± 6.80
Peak LVP (mmHg)	107.70	114.60	107.01	109.77 ± 4.20

### Model Calibration

**Figure 4:**
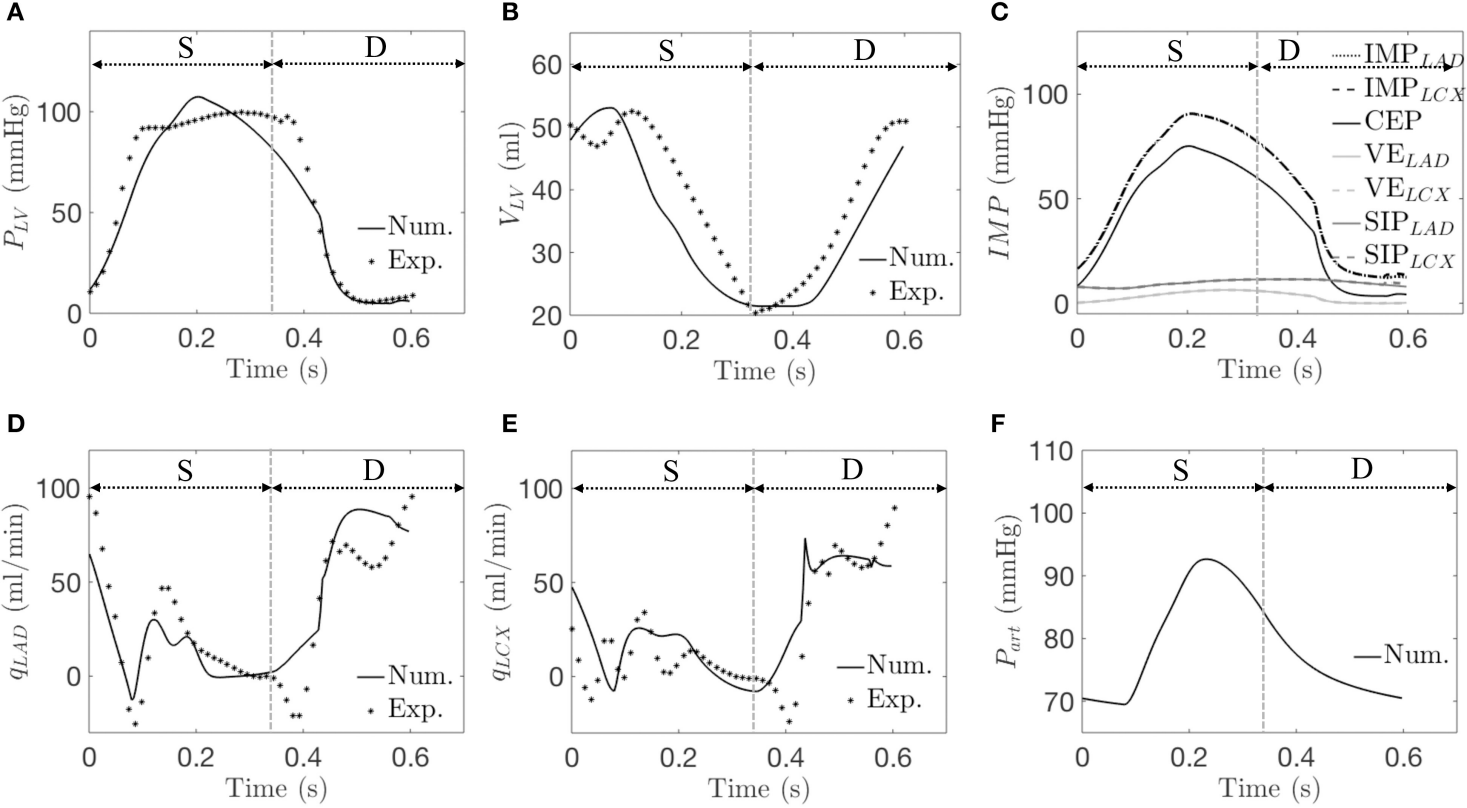
A representative simulation is compared with data from the control group. Experimentally (Exp.) and numerically (Num.) predicted waveforms of **(A)** LV pressure; **(B)** LV volume; **(C)** *IMP* and its three components (Num. only); **(D)** *q*_*LAD*_; **(E)** *q*_*LCX*_; and **(F)** arterial pressure (Num. only). “S” and “D” denote systole and diastole, respectively.

The arterial pressure, LV pressure, LAD, and LCX flow rates, and LV volume predicted by the simulations are in agreement with the experimental measurements (see Table 2 and a representative case in Figure 4). The average error between the model predictions and measurements for the three animals for ∑ *Q*_*LAD*_, ∑ *Q*_*LCX*_, LV EDV, and peak LVP are 0, 0, 0.4, and 0.2%, respectively. While there are some slight discrepancies in the model predicted time-course (e.g., in the LV volume waveform and the magnitude of the first up-shoot during systole in the LAD coronary flow rate waveform) when compared to the experimental measurements, the calibrated model, nevertheless, can reproduce key features of the measured LV volume, pressure and coronary flow rate waveforms (Figures 4D,E). Specifically, the model predicted a reduced coronary flow rate in the LCX and LAD networks during early systole (*t* = 0–0.06 s) because as *IMP* starts to increase, *P*_*art*_ has yet to increase during this period of time (Figures 4A,F). The flow rate then oscillates (*t* = 0.06–0.33 s) as the perfusion pressure, which is largely driven by *P*_*art*_, rises and competes with *IMP* to control the flow. With the decay of *IMP* during diastole (*t* > 0.33 s), the perfusion pressure dominates, and the flow rate increases. Three components of *IMP* associated with the myocardium-vessel interaction mechanism show that the contribution of each mechanism is different (i.e., mean CEP, ~76%, mean SIP, ~18% and mean VE, ~6%) (Figure 4C). We note that since the LCX territory (LVFW) and LAD territory (LV septum) contract synchronously in the control simulations, they both have the same *IMP* waveforms (i.e., *IMP*_*LAD*_ = *IMP*_*LCX*_) due to their identical VE and SIP components.

### Isolated Mechanical Dyssynchrony

**Figure 5:**
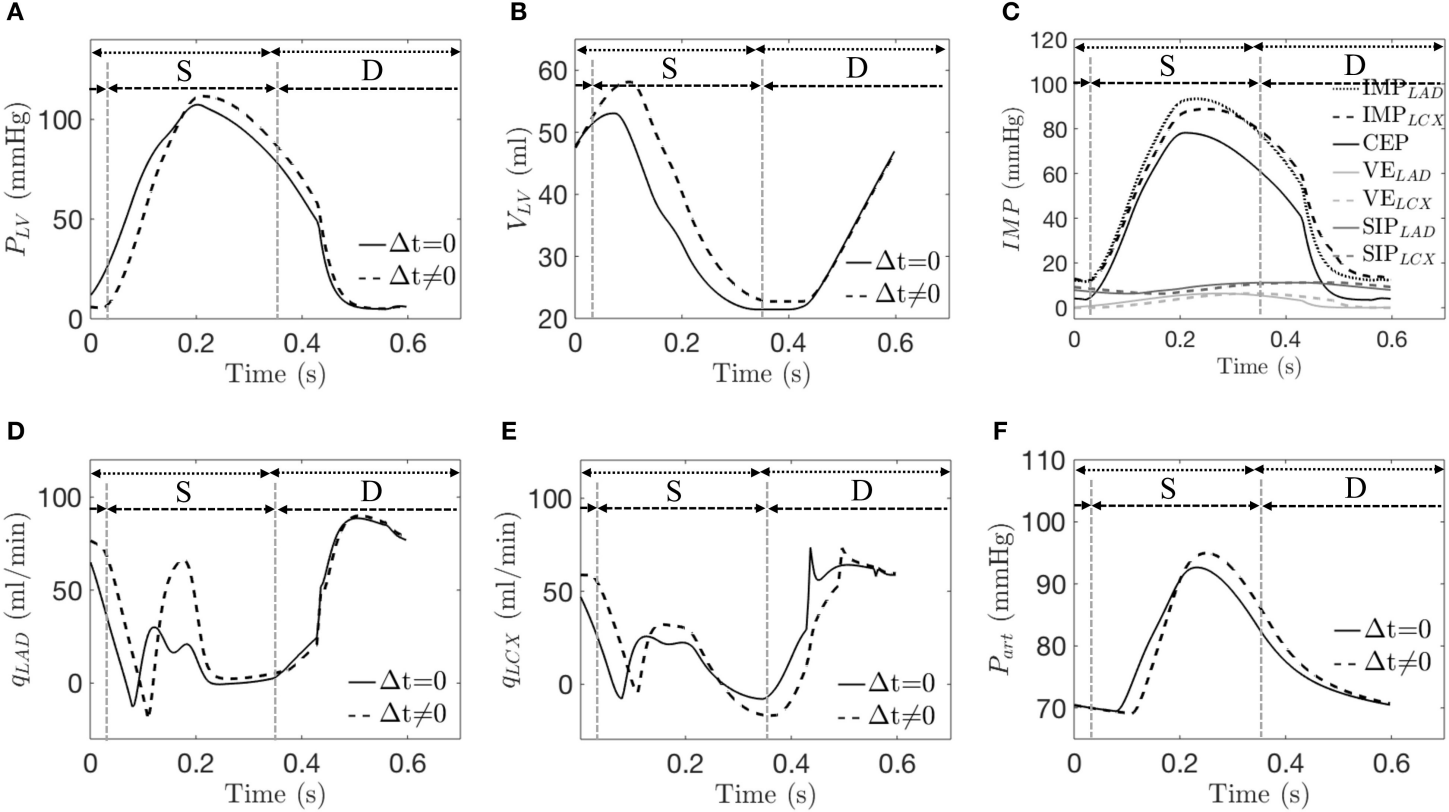
A representative simulation of the control case (Δ*t* = 0) and isolated mechanical dyssynchrony case (*t* ≠ 0 with SDI = 10%). Comparison of **(A)** LV pressure; **(B)** LV volume; **(C)** *IMP* and its three components at the LAD and the LCX for only in isolated mechanical dyssynchrony; **(D)** *q*_*LAD*_; **(E)** *q*_*LCX*_; and **(F)** arterial pressure. Dotted and dash lines denote a control and an isolated dyssynchrony with both systole (S) and diastole (D).

From the simulations for isolated mechanical dyssynchrony (SDI > 0), the waveforms for the LV pressure, LV volume, LCX flow rate, and arterial volume are all shifted to the right compared to the control simulations (SDI = 0) as a result of a time delay, Δ*t* in the contraction of the LCX territory with respect to the LAD territory (Figures 5A,B,E,F). The LAD flow rate waveform is, however, less shifted (Figure 5D). The model predicted a larger EDV because the delayed contraction causes a delay in the rise of the LV pressure, which in turn results in a delay of the onset of end-diastole. Because of the larger EDV, peak *P*_*LV*_ is increased (based on Equation 15). Mean value of *P*_*LV*_ is, however, reduced compared to that of the control group because of the delay in the rise of *P*_*LV*_ during systole (Figure 5A). As a result, the peak value of the CEP component increases but its mean value decreases (Figure 5C). On the other hand, the VE_*LAD*_ waveform remains synchronous with the CEP waveform while the VE_*LCX*_ waveform is dyssynchronous but with the same magnitude. Correspondingly, the peak *IMP*_*LAD*_ is greater while the peak *IMP*_*LCX*_ is smaller than the corresponding values in the control simulations (Figures 4C, 5C). Because of the wave shift of *IMP*s and perfusion pressure that is largely driven by *P*_*art*_ (during *t* = 0−0.12*s*), there is a corresponding wave shift in terms of *q*_*LAD*_ and *q*_*LCX*_ in which the first up shoot during systole is delayed by ~0.07s (Figures 5D,E) Due to the greater increase of *P*_*art*_ than *IMP*_*LAD*_ during *t* = 0.12 − 0.22 *s*, coronary flow in the LAD increases in the isolated mechanical dyssynchrony simulations. While due to the decay of peak *IMP*_*LCX*_ resulted from the time delay, *q*_*LCX*_ decreases in late systole and early diastole, resulting in a reduction of the LCX coronary flow in the isolated mechanical dyssynchrony simulations.

**Figure 6:**
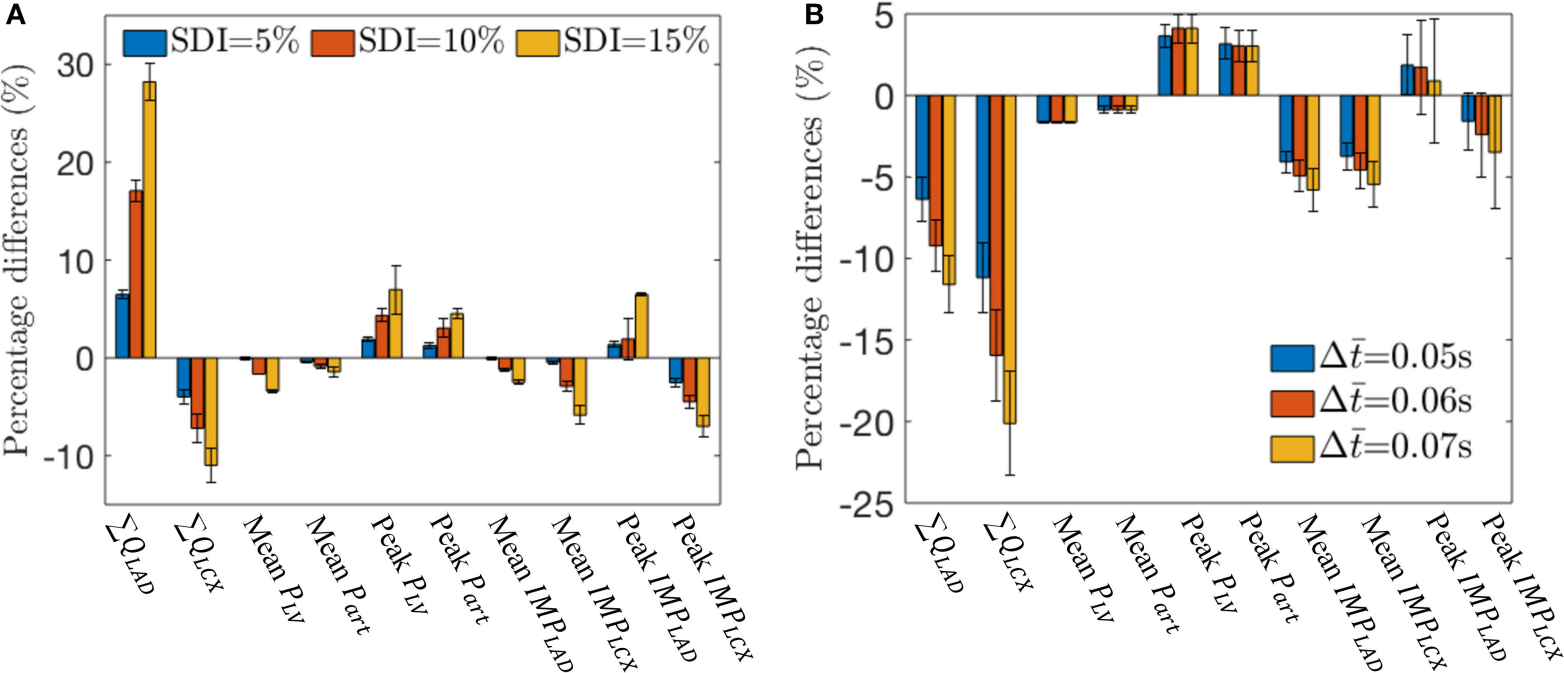
Effects of SDI on total coronary flow, mean arterial and LV pressures, and *IMP*_*LAD*_ and *IMP*_*LCX*_ over three swine. **(A)** Percentage difference of the quantities with respect to the control simulation for different SDI. **(B)** Percentage difference of the quantities with respect to the control simulation for different $\Delta \bar{t}$ with SDI = 10%.

These effects of mechanical dyssynchrony on *P*_*art*_, *P*_*LV*_, *q*_*LAD*_, *q*_*LCX*_, *IMP*_*LAD*_, and *IMP*_*LCX*_ are all consistent in the animal-specific simulations of the three swine models. Coronary flow and *IMP* in the LAD and LCX, as well as the mean and peak values of *P*_*LV*_ and *P*_*art*_ all vary monotonically with increasing degree of mechanical dyssynchrony as SDI increases from 5 to 15% (Figure 6A). Specifically, ∑ *Q*_*LAD*_, peak *P*_*LV*_, peak *P*_*art*_, and peak *IMP*_*LAD*_ increase with SDI while all the other quantities decrease with SDI. We note that peak *IMP*_*LAD*_ increases while the mean *IMP*_*LAD*_ decreases with increasing SDI as explained earlier. Therefore, the disparity between LAD and LCX flows increases with increasing SDI. For an SDI of 15%, ∑ *Q*_*LAD*_ is increased by about 28% whereas ∑ *Q*_*LCX*_ is decreased by about 12% compared to control simulation (Figure 6A). Given that the coronary flow rate is a competition between perfusion pressure and *IMP*, the percentage change in mean *P*_*art*_ (~1% at SDI = 15%) relative to that of the *IMP*s (~3% in LAD and ~6% LCX at SDI = 15%) at a fixed SDI in mechanical dyssynchrony implies that the changes in coronary flow rates are affected more by the changes in regional *IMP*s than the perfusion pressure.

With a time delay $\Delta \bar{t}$ prescribed to the VE and CEP components of both the *IMP*_*LAD*_ and *IMP*_*LCX*_ in Equation (21), the simulation shows that at an SDI = 10%, LAD flow is reduced (Figure 6B). This is because *IMP*_*LAD*_ is delayed with respect to *P*_*LV*_ with $\Delta \bar{t}>0$, which reduces the flow. With Δ$\bar{t}>0$, there is more delay in *IMP*_*LCX*_, which impedes more flow during early diastole. Increasing the time delay $\Delta \bar{t}$ also reduces the peak *IMP*_*LAD*_ as VE_*LAD*_ and CEP becomes asynchronous. With $\Delta \bar{t}=0.07$s, ∑ *Q*_*LAD*_ and ∑ *Q*_*LCX*_ are decreased by 12 and 20% with respect to the control simulations (Figure 6B).

### Mechanical Dyssynchrony With Ischemia

**Figure 7:**
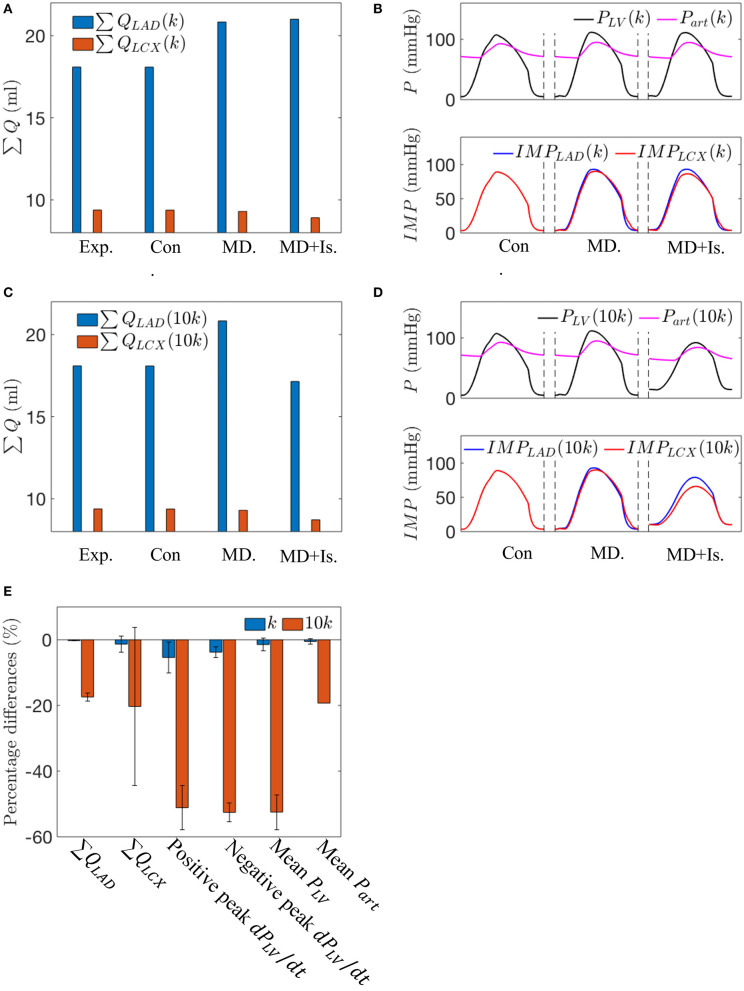
A comparison between the simulations for the control case, the isolated mechanical dyssynchrony case, and the mechanical dyssynchrony (SDI = 10%) + ischemia case for *k* = 0.34 and 10*k* = 3.40 mmHg/ml^2^ for the following variables: **(A)** Coronary flow in the LAD and LCX with *k*; **(B)** Arterial Pressure, LV pressure waveforms and *IMP*s with *k*; **(C)** Coronary flow in the LAD and LCX with 10*k*; **(D)** Arterial Pressure, LV pressure waveforms and *IMP*s with 10*k*; and **(E)** Percentage differences over three swine using different slopes (k) in the contractility-flow relationship in ischemic region of LCX in terms of flow, *dP*_*LV*_/*dt* and mean arterial and LV pressures between the isolated mechanical dyssynchrony and mechanical dyssynchrony + ischemia cases.

In the mechanical dyssynchrony with ischemia (MD + IS) simulations for an SDI = 10%, both *E*_*es,LAD*_ and *E*_*es,LCX*_ are prescribed as a function of ∑ *Q*_*LAD*_ and ∑ *Q*_*LCX*_, respectively, based on a contractility-flow relationship (Figure 3). The gradients in ischemic regions (*k*_*LAD*_, *k*_*LCX*_) were obtained by taking the ratio of the maximal chamber elastance under control condition (*E*_*es*_ = 3.18) with the experimentally measured coronary flow (∑ *Q*_*LAD, n*_, ∑ *Q*_*LCX, n*_) for each swine (Table 2). The values of (*k*_*LAD*_, *k*_*LCX*_) for the three swine models are (0.18, 0.34), (0.27, 0.38), and (0.17, 0.22) mmHg/ml^2^. Coupling of the contractility with coronary flow for these values of *k* produced only a small decrease in ∑ *Q*_*LAD*_ (0.21%) and ∑ *Q*_*LCX*_ (4.1%) when compared to the isolated mechanical dyssynchrony case (Figure 7A). For these values of *k*, coupling of the contractility with coronary flow, which changes during mechanical dyssynchrony, did not produce substantial changes in peak *P*_*LV*_, *P*_*art*_, and *IMP*_*LAD*_. The coupling produced only a slightly reduced *IMP*_*LCX*_ (4.1%) compared to that in the isolated mechanical dyssynchrony case (Figure 7B). The *IMP*_*LCX*_ is reduced because of a reduction in ∑ *Q*_*LCX*_ (4.1%) in mechanical dyssynchrony that decreases *E*_*es,LCX*_ (contractility in LCX), which in turn reduces the VE component of *IMP*_*LCX*_.

Increasing the gradient *k* in the ischemic regime of the contractility-flow relationship for LCX by 10 times produced pronounced changes in the hemodynamics (Figures 7C,D). A steeper contractility-flow relationship produced a substantial reduction in *E*_*es,LCX*_, which decreases the overall LV contractility and led to a substantial reduction in *P*_*LV*_ and *P*_*art*_ (Figure 7D) that in turn significantly decreases flows to the LAD and LCX territory (Figure 7C). The decrease in LAD and LCX flows further reduced the overall LV contractility until the pressure waveforms reached a steady periodic state. Averaging the effects of incorporating the different contractility-flow relationships in the three swine models (Figure 7E), we find that the resultant maximum rate of LV pressure rise (positive and negative *dP*_*LV*_/*dt*) which is an index of contractility is reduced by 45–55% in mechanical dyssynchrony with ischemia when *k* is large.

## Discussion

The key findings of this study are: (1) an increase in time delay Δ*t* (SDI) in the *IMP*_*LCX*_ waveform with respect to *IMP*_*LAD*_, LV, and arterial pressure waveforms produces a decrease in LCX flow and an increase in LAD flow; (2) a time delay $\Delta \bar{t}$ in both *IMP*_*LAD*_ and *IMP*_*LCX*_ waveform with respect to the LV and arterial pressure waveforms causes both LAD and LCX coronary flow to decrease; (3) prescribing a direct relationship between regional contractility and its corresponding coronary flow (Figure 3) can affect global LV mechanics, hemodynamics and function, which in turn further affects the coronary flow in a feedback loop in mechanical dyssynchrony, especially when contractility is highly sensitive to flow (i.e., large gradient *k* in the ischemic regime of the relationship). These findings imply that during mechanical dyssynchrony, regional *IMP* plays a significant role in affecting regional coronary flows, which can then affect global LV function in a feedback loop that produces ischemia if contractility is sensitive to the coronary flow changes. Correspondingly, correcting for mechanical dyssynchrony by CRT may help restore regional *IMP* waveform and coronary flow that improve both regional and global LV contractility.

We developed a closed-loop cardiac-coronary modeling framework consisting of the systemic and the coronary circulations to interrogate the bi-directional interactions between LV mechanics and coronary flow in mechanical dyssynchrony. The modeling framework was calibrated using the LV pressure and volume, LAD and LCX flow rate waveforms that were measured experimentally in three (normal) RA pacing swine models. We then applied the calibrated framework to investigate the isolated effects of mechanical dyssynchrony on coronary flow, as well as the effects of mechanical dyssynchrony when regional coronary flow and contractility are coupled. We show that regional coronary flows in the LCX and LAD are altered by asynchronous LV contraction due to changes in the arterial pressure, LV pressure, *IMP*_*LAD*_ and *IMP*_*LCX*_ waveforms as discussed below.

We show that a delay or phase shift in the *IMP*_*LCX*_ waveform with respect to the *IMP*_*LAD*_ waveform that is synchronous with the LV and arterial pressures produces a reduction in LCX flow, but an increase in LAD flow. To produce a reduction in LAD flow (in addition to a reduction in LCX flow) requires a delay $\Delta \bar{t}\;$or phase shift in *IMP*_*LAD*_ with respect to the LV and arterial pressures. These model predictions are consistent with experimental studies, which have shown that in RV pacing or LBBB (Ono et al., 1992; Skalidis et al., 1999, 2001; Kyriakides et al., 2007), regional myocardial blood flow in the septum is significantly reduced compared to the reduction in LVFW after LBBB induction. Studies on canine model with pacing at different ventricular sites (Delhaas et al., 1994; Amitzur et al., 1995) also show a significant reduction in regional blood flow in the early activated regions compared to the late activated regions. Specifically, Amitzur et al. (1995) shows that RV pacing causes a reduction in LAD flow without any change in the LCX flow when perfusion pressure is fixed (17–45%) or not fixed (~10%) as compared to RA pacing (representing normal activation). Under maximal vasodilation using adenosine, however, they reported no changes in LAD flow with RV pacing. In contrast to that study, a few clinical studies (Skalidis et al., 2001; Itoh et al., 2015) show that flow (as measured by flow velocity time integral) is reduced at maximal vasodilation in both the LCX and LAD arteries in LBBB patients compared to when they are treated with CRT (Itoh et al., 2015) or to control subjects (Skalidis et al., 2001). This discrepancy between the experimental and clinical studies may be attributed to anatomical differences in the coronary vasculature between humans and canine (Vernooy et al., 2007). Our model prediction that both LAD and LCX flow changes with mechanical dyssynchrony is consistent with clinical studies (Skalidis et al., 2001; Itoh et al., 2015).

Because mechanical dyssynchrony produces regional changes in mechanics (e.g., regional myocardial fiber strain, work done and wall tension) leading to a redistribution of myocardial oxygen demand (Prinzen et al., 1999) [e.g., RV pacing is found to reduce and increase mechanical work in the septum and the LVFW, respectively (Prinzen et al., 1990, 1999)], some studies have hypothesized that the regional alteration in coronary flow found in mechanical dyssynchrony is caused by regional changes in metabolic demand (Prinzen et al., 1990). These alterations, however, persist even under hyperemia or maximal vasodilation in the absence of autoregulation mechanisms (i.e., metabolic, myogenic, and shear regulations) (Prinzen et al., 1990) as described in the above studies. Indeed, a study (Itoh et al., 2015) has shown that both LAD and LCX flows are moderately reduced by about 14 and 6% in asynchronous activation under hyperemia, which are within the range of reductions predicted by our model when *IMP*_*LAD*_ is delayed by about $\Delta \bar{t}>0.05\;s$ (Figure 6B). These findings further suggest that regional changes in *IMP* and perfusion pressure play a significant role on the regional changes in coronary flow during mechanical dyssynchrony. The mechanisms of *IMP* are attributed to the contraction of the myocyte (by the changing of myocardial tissue elasticity and shortening induced pressure) and the cavity pressure (Algranati et al., 2010). Some studies (Skalidis et al., 1999, 2001; Kyriakides et al., 2007) suggest that asynchronous activation due to RV pacing or LBBB causes an increase in *IMP* in the interventricular septum that reduces myocardial perfusion but the effects on *IMP*_*LCX*_ has not been measured. Here, in our model prediction, the regional variation of LV mechanics in mechanical dyssynchrony produces regional changes in *IMP* (*IMP*_*LAD*_ increases and *IMP*_*LCX*_ decreases) that in turn alters the coronary flow. Taken together with the clinical and experimental studies mentioned above, our finding underscores the significance of the regional *IMP* in regulating the regional coronary flow during mechanical dyssynchrony.

Because myocardial tissue contractility depends on coronary flow (Ross, 1991), we hypothesized that regional variation in coronary flow can in turn affect the regional contractility, especially when coronary flow is impeded. To interrogate this effect, we prescribed (for the first time) a regional contractility-flow relationship (*E*_*es*_ − ∑ *Q*) in the modeling framework based on experimental observations (Ross, 1991). The model predicts that the reduction in LCX coronary flow during mechanical dyssynchrony led to a decrease in *E*_*es,LCX*_ that affects LV and arterial pressures as well as *IMP*_*LAD*_ and *IMP*_*LCX*_, causing further effects on the coronary flow. Since the contractility is highly sensitive to the coronary flow in the ischemic regime (i.e., large gradient *k*) of the contractility-flow relationship, a small reduction in the regional coronary flow in mechanical dyssynchrony leads to a large decrease in the corresponding regional and overall LV contractility as indicated by a reduction in *dP*_*LV*_/*dt*, a metric associated with myocardial contractility (Theroux et al., 1974). The reduction in overall LV contractility in turn reduces the perfusion pressure and regional *IMP* that further reduces coronary flow in a feedback loop. These results suggest that mechanical dyssynchrony can produce ischemia (i.e., a reduction in global LV contractility due to a reduction in regional coronary flow), especially when contractility-flow relationship has a steep gradient. Existing evidence of ischemia caused solely by asynchronous activation from previous studies are conflicting (Kuhn et al., 1982; Ono et al., 1992). While an experimental study has reported no change in lactate production with the induction of LBBB in a canine model at resting heart rate (Ono et al., 1992), a clinical study measuring lactate production in the myocardium in 95 patients with no coronary disease and a normal coronary angiogram has suggested that mechanical dyssynchrony may induce myocardial ischemia at a high-rate of pacing (Kuhn et al., 1982). The latter study is supported by the findings that LBBB patients often complain of exertional dyspnea despite normal left ventricular dimensions and function at rest, which might imply the induction of ischemia (Breithardt and Breithardt, 2012). Although we have not taken into account coronary autoregulation due to changes in metabolic demand that should be present in these studies (especially at higher heart rate), our model suggests that the local changes in coronary flow may produce global changes in LV function that is indicative of ischemia (i.e., reduced *dP*_*LV*_/*dt*). This is especially so when the myocardial contractility is very sensitive to flow conditions, which may occur when metabolic regulation is exhausted.

While modeling of coronary perfusion using the poroelastic theory in a finite element framework (Chapelle et al., 2010; Lee et al., 2016) and 1D Navier-Stokes equation (Smith et al., 2002) can take into account more details associated with coronary flow (e.g., myocardial tissue microstructure, geometrical variation in the tissue, wave reflection, etc.), lumped models of the coronary vasculature (such as this work) (Wang et al., 1989; Duanmu et al., 2018; Namani et al., 2020) sacrifice these details for computational efficiency in simulating mechanical and hemodynamic changes associated with heart diseases. To the best of our knowledge, we have, for the first time, bi-directionally coupled the coronary circulation with LV mechanics in a closed-loop manner to model the effects of mechanical dyssynchrony on both the LV's perfusion and its mechanics. Bi-directional interactions between the regional LV mechanics and the coronary vasculature in this novel modeling framework occur both directly (*IMP* affects flow and flow affects contractility) and indirectly (LV mechanics affects perfusion pressure that in turn affects flow). By dividing the LV into two compartments associated with the LAD and LCX regions based on the idea from Sunagawa et al. (1983), we were able to simulate the corresponding regional changes in coronary flow associated with asynchronous activation of the perfused territories. By prescribing a contractility-flow relationship in the LAD and the LCX regions, we were also able to simulate, for the first time, how those changes affect the regional LV contractility that in turn affects the LV hemodynamics globally in a feedback loop. The calibrated flow rate waveform in the LAD predicted by our model (0D) (Figure 4A) is also comparable with the predictions of other published coronary flow models (Liang et al., 2007; Mynard et al., 2014; Duanmu et al., 2018), including models based on the 1D Navier-Stokes equation (refer to Appendix F for the comparison). Given these capabilities, our model offers a valuable and efficient approach to understand the mechanisms that affect coronary flow in mechanical dyssynchrony that maybe useful for applying CRT in heart failure. In particular when taken together with the consistent observation that LVFW mechanical work is increased in mechanical dyssynchrony, our model predictions imply the presence of a stronger compensatory control mechanism to increase (or at least maintain) coronary blood flow to the LVFW, further suggesting that defects in autoregulation may play a central role in discriminating CRT responder from non-responder.

### Limitations

There are some limitations associated with this study. First, we did not consider the autoregulation mechanism in coronary flow analysis. As such, the flow predicted by the model is only driven by perfusion pressure and *IMP* under passive vessel's condition. Coronary autoregulation has been shown to maintain blood flow relatively constant across a wide range of perfusion pressure (Johnson, 1986; Namani et al., 2018a). This mechanism can be incorporated into the existing framework in future studies to investigate the role of autoregulation in asynchronous activation. Second, 0D lumped model used in this study is able to predict the main features of coronary flow rate waveforms but unable to capture the wave oscillations. This issue can be addressed by coupling with a 1D model. Third, LV mechanics is simplified using a time-varying elastance model by a rule-of-mixture based on the approach by Sunagawa et al. (1983). Although this approach is able to represent key characteristics of the LV, it does not take into account detailed mechanical properties (e.g., anisotropy), microstructure of the LV (e.g., myofiber orientation) and the length-dependent effects in the cardiac muscles. These limitations may account for some of the discrepancies between model predicted and experimentally measured LV pressure and volume waveforms in terms of the time course. Nevertheless, we have shown that the model predictions are consistent with the experimental results in reproducing key features of the waveforms. This limitation can be overcome by using a finite element model for describing LV mechanics (Arumugam et al., 2019). Fourth, we have not included the RV in the modeling framework. Although the RV pressure is small compared to the LV, it may affect the cavity pressure component of the *IMP* in the LAD territory, which was assumed to be the same as in the LCX territory here. To address this issue, we have performed a sensitivity analysis with a different CEP component in the LAD that considers the effects of RV pressure (refer to Appendix G for sensitivity analysis). The analysis show that the conclusion is not altered when we consider the effects of RV pressure in the LAD *IMP*. The effects of RV on *IMP* can be investigated in greater detail in future studies using finite element modeling. Fifth, because the goal here is not to infer and interpret any changes in the model parameters arising from the calibration of measurements associated with the experimental/clinical data, we did not perform a rigorous sensitivity analysis of all the parameters and uncertainty quantification analysis. Such analyses will be performed in future studies. Last, model predictions of RV pacing were compared with experimental data from previous published studies as mentioned in the discussion, but not with animal-specific measurements. This can be addressed in future studies with animal-specific measurements made under RV and RA pacing.

## Conclusion

In summary, we have developed and calibrated a closed-loop cardiac-coronary modeling framework that takes into account the bi-directional interaction between LV mechanics, systemic circulation and coronary perfusion. We applied the modeling framework to simulate mechanical dyssynchrony and show that LV mechanics and coronary flow are affected by asynchronous contraction and interacts bi-directionally. We show that the changes in coronary flow are sensitive to a phase shift in the regional *IMP* waveform that may arise from asynchronous activation. We further show that, depending on the regional contractility-flow relationship, the altered regional coronary flow can influence the global LV hemodynamics and regional LV mechanics that in turn further affects the coronary flow to induce myocardial ischemia.

## Data Availability Statement

The raw data supporting the conclusions of this article will be made available by the authors, without undue reservation.

## Ethics Statement

The animal study was reviewed and approved by the Institutional Animal Care and Use Committee.

## Author Contributions

LF, RN, GK, and LL contributed to the conception and design of the study. JC contributed to the conduction experimental procedure. LF, RN, JC, GK, and LL contributed to the analysis and interpretation of data, the drafting of the manuscript, revising it critically for important intellectual content, and final approval of the manuscript. All authors have significantly contributed to the submitted work.

## Conflict of Interest

The authors declare that the research was conducted in the absence of any commercial or financial relationships that could be construed as a potential conflict of interest.
