# Supplementary material for: Effects of Mechanical Dyssynchrony on Coronary Flow: Insights From a Computational Model of Coupled Coronary Perfusion With Systemic Circulation
Source: Front Physiol. 2020 Aug 14;11:915. doi: 10.3389/fphys.2020.00915 (PMC7457036; doi:10.3389/fphys.2020.00915)
Supplement: Supplementary file 1 [file Presentation_1.pdf]

**APPENDIX A**: LV PRESSURE

As the pressure in left ventricle (LV) is assumed to be the same as the pressure in the LAD and LCX in any cases, we have $P_{LV} = P_{LAD} = P_{LCX}$. In this model, the pressure in the LAD compartment in terms of $V_{LV,LAD}$ is

$$P_{LV} = P_{LAD} = \frac{E_{es,LAD}\, e(t)}{F}\left(V_{LV,LAD} - V_{LV0,LAD}\right) + (1 - e(t))P_{ed}, \tag{A1}$$

where $F$ is the prescribed fraction of the LAD volume of the total LV volume. The pressure in the LCX compartment in terms of $V_{LV,LCX}$ is

$$P_{LV} = P_{LCX} = \frac{E_{es,LCX}\, e(t+\Delta t)}{1-F}\left(V_{LV,LCX} - V_{LV0,LCX}\right) + \left(1 - e(t+\Delta t)\right)P_{ed}. \tag{A2}$$

Rearrange Eq. (A1) and Eq. (A2), we have

$$(P_{LV} - (1 - e(t))P_{ed})\frac{F}{E_{es,LAD}\, e(t)} = \left(V_{LV,LAD} - V_{LV0,LAD}\right), \tag{A3}$$

and

$$(P_{LV} - (1 - e(t+\Delta t))P_{ed})\frac{1-F}{E_{es,LCX}\, e(t+\Delta t)} = \left(V_{LV,LCX} - V_{LV0,LCX}\right). \tag{A4}$$

Add Eq. (3) and (A4), we have

$$\frac{P_{LV}FE_{es,LCX}\, e(t+\Delta t) + P_{LV}(1-F)E_{es,LAD}\, e(t)}{E_{es,LAD}\, e(t)E_{es,LCX}\, e(t+\Delta t)} \tag{A5}$$
$$= (V_{LV} - V_{LV0}) + \frac{F\left(1 - e(t)\right)P_{ed}}{E_{es,LAD}\, e(t)} + \frac{(1-\ F)\left(1 - e(t+\Delta t)\right)P_{ed}}{E_{es,LCX}\, e(t+\Delta t)}$$

Then the LV pressure in terms of the total LV volume is given by rearranging Eq. (A5) as

$$P_{LV} = \left[(V_{LV} - V_{LV0}) + \frac{F\left(1 - e(t)\right)P_{ed}}{E_{es,LAD}\, e(t)} + \frac{(1-F)\left(1 - e(t+\Delta t)\right)P_{ed}}{E_{es,LCX}\, e(t+\Delta t)}\right]\frac{E_{es,LAD}\, e(t)E_{es,LCX}\, e(t+\Delta t)}{FE_{es,LCX}\, e(t+\Delta t)+(1-F)E_{es,LAD}\, e(t)}. \tag{A6}$$

**APPENDIX B**: PARAMETER SETS OBTAINED FROM CALIBRATING THE LV-SYSTEMIC CIRCULATION MODEL

Using the lumped model of the LV-Systemic circulation, parameter sets for three swine including the resistances, capacitances, resting values and initial guesses of LA and LV volumes are listed. Values for each parameter marginally vary between swine as hemodynamic behavior is animal specific (**Table B1**).

**Table B1**: Parameters used in lumped model for three swine.

| Parameter | Unit | 1 | 2 | 3 |
|---|---|---|---|---|
| $R_{ao}$ | mmHg s/ml | $1.00 \times 10^{-1}$ | $3.50 \times 10^{-1}$ | $1.00 \times 10^{-1}$ |
| $R_{ven}$ | mmHg s/ml | $7.25 \times 10^{-2}$ | $7.25 \times 10^{-2}$ | $7.25 \times 10^{-2}$ |
| $R_{mv}$ | mmHg s/ml | $4.44 \times 10^{-1}$ | $4.44 \times 10^{-1}$ | $4.44 \times 10^{-1}$ |
| $R_{per}$ | mmHg s/ml | $3.00 \times 10^{-1}$ | $3.00 \times 10^{-1}$ | $3.00 \times 10^{-1}$ |
| $R_{LAD}$ | mmHg s/ml | $1.59 \times 10^{-1}$ | $5.70 \times 10^{-3}$ | $2.54 \times 10^{-1}$ |
| $R_{LCX}$ | mmHg s/ml | 2.92 | 1.93 | 1.10 |
| $C_{ven}$ | ml/mmHg | 7.60 | 7.60 | 7.60 |
| $C_{art}$ | ml/mmHg | $4.00 \times 10^{-1}$ | $3.20 \times 10^{-1}$ | $4.00 \times 10^{-1}$ |
| $V_{art0}$ | ml | 10 | 0 | 10 |
| $V_{ven0}$ | ml | 20 | 20 | 20 |
| $V_{LV0}$ | ml | -7 | -10 | -7 |
| $V_{LA0}$ | ml | 0 | 0 | 0 |
| $V_{art}$ | ml | 690 | 650 | 830 |
| $V_{ven}$ | ml | 0 | 0 | 0 |
| $V_{LV}$ | ml | 15 | -25 | 15 |
| $V_{LA}$ | ml | 0 | 0 | 0 |

## **APPENDIX C**: DETERMINATION OF $\alpha$ AND $\beta$

In the formulation of *IMP* (see Eq. (21)), a parameter set ($\alpha$ and $\beta$) has to be determined based on some requirements. The selected $\alpha$ and $\beta$ should be able to 1) best match the experimentally measured flow rate waveforms; 2) best match the experimentally measured total coronary flow; 3) guarantee that CEP and VE contributions are consistent with previous experimental observations as discussed in the main text. In both LAD and LCX territories, it can be observed that different sets of $\alpha$ and $\beta$ values in the *IMP* function predict different flow rate waveforms (**Figure C1a-C1b)** with different total coronary flow. The percentage differences between model predicted and experimentally measured total coronary flow for the first swine model using different sets of $\alpha$ and $\beta$ in both LAD and LCX are given (**Figure C1c-C1d**). In order to find a set of $\alpha$ and $\beta$, which can better satisfy the three requirements mentioned above, a non-dimensional residual variable $J$ is defined as

$$J = \left\{\left(\frac{\sum Q_N - \sum Q_E}{\sum Q_E}\right)^2 + \sum_{i=1}^{n}\left(\frac{q_N^i - q_E^i}{q_E^i}\right)^2 + \left(\frac{VE+SIP-30\%\times IMP}{30\%\times IMP}\right)^2\right\}^{\frac{1}{2}} \quad \text{(C1)}$$

for LAD and LCX. In Eq. (C1), "N" and "E" denote numerical predictions and experimental measurements, $q_N^i$ is the flow rate at *i*th time point and n is the total number of time points in one cardiac cycle. Since different parameter sets of $\alpha$ and $\beta$ have different residual number $J_{LAD}$ and $J_{LCX}$ corresponding to the LAD and LCX, the parameter set associated with minimum $J$ is selected (**Figure C1e-C1f**).

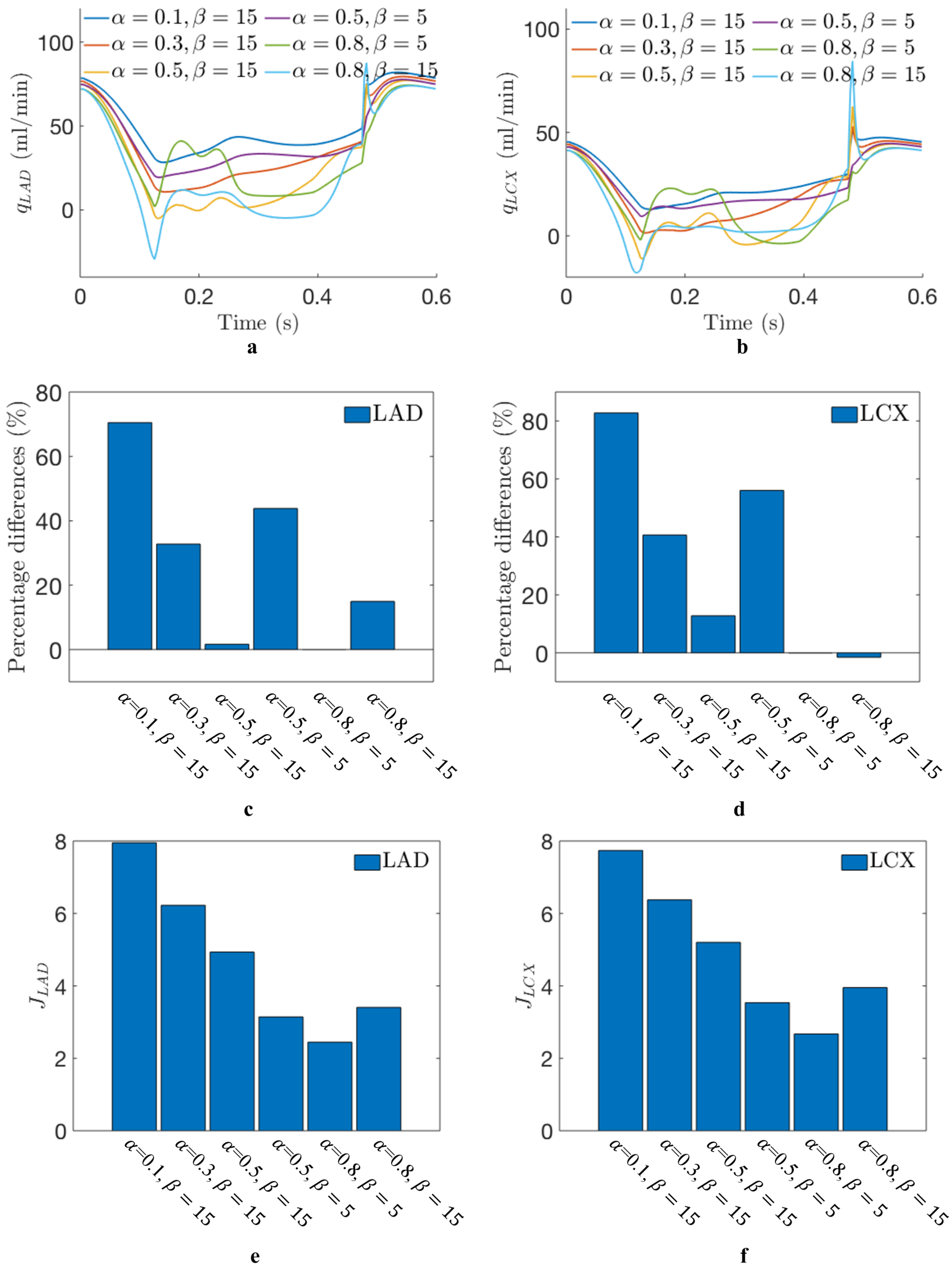


**Figure C1**: Coronary flow rate waveforms, percentage differences of flow between experimental data and model prediction, and residual variable $J$ with different $\alpha$ and $\beta$: **a.** flow rate waveform in the LAD; **b.** flow rate waveform in the LCX; **c.** percentage difference in the LAD; **d.** percentage difference in the LCX; **e.** residual value in the LAD; **f.** residual value in the LCX.

**APPENDIX D**: THE COMPARISON OF FLOW RATE WAVEFORMS PREDICTED BY THE MODEL IN THIS STUDY AND IN LITERATURE

A comparison of the flow rate waveforms with different coronary flow models is given in **Figure D1.** All the flow rate waveforms have been scaled so that they have the same total coronary flow over a cardiac cycle and heart rate as the representative case (**Figure 4**). There is an overall qualitative agreement between our simulations and the published simulation results in capturing the systolic and diastolic flow transients. However, our prediction is less wavy during late diastole ($t = 0.45 - 0.6s$) compared to Mynard's(Mynard et al., 2014) data, due to the fact that they used a combined 0D and 1D model for coronary flow analysis in the newborn lamb with more prominent wave propagation ("oscillations"). The same combined 0D and 1D model was used by Ge(Liang et al., 2007), but their predicted flow rate waveform is under autoregulated conditions.

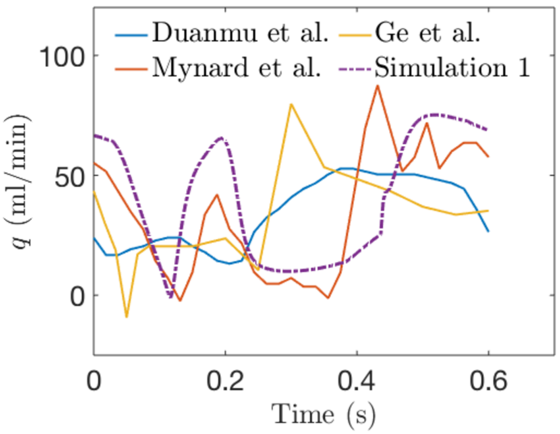


**Figure D1**: Coronary flow rate waveforms predicted by different computational models.

**APPENDIX E**: THE EFFECTS OF RV PRESSURE IN $IMP_{LAD}$ ON CORONARY FLOW, ARTERIAL AND LV PRESSURES, AND $IMP$S IN MECHANICAL DYSSYNCHRONY

Since the LAD territory supplies a part of the septum and the LCX territory is mostly the LVFW, the LAD and LCX territories have different mechanisms for CEP component in $IMP$, namely CEP= $P_{LV} - P_{RV}$ and CEP= $P_{LV}$, respectively, with an assumed $P_{RV}$ of $0.2P_{LV}$ (Mynard et al., 2014). In order to show the effects of mechanical dyssynchrony with considering RV pressure in $IMP_{LAD}$, we ran two sets of simulations with and without considering the RV pressure in $IMP_{LAD}$ for one swine model and perform the sensitivity analysis in terms of SDI. The comparison of the percentage difference of the quantities with different SDI between control and mechanical dyssynchrony cases is given in **Figure E1.** Although the magnitude of the change is different between simulations with and without considering the RV pressure, the trends for each quantity remain unchanged.

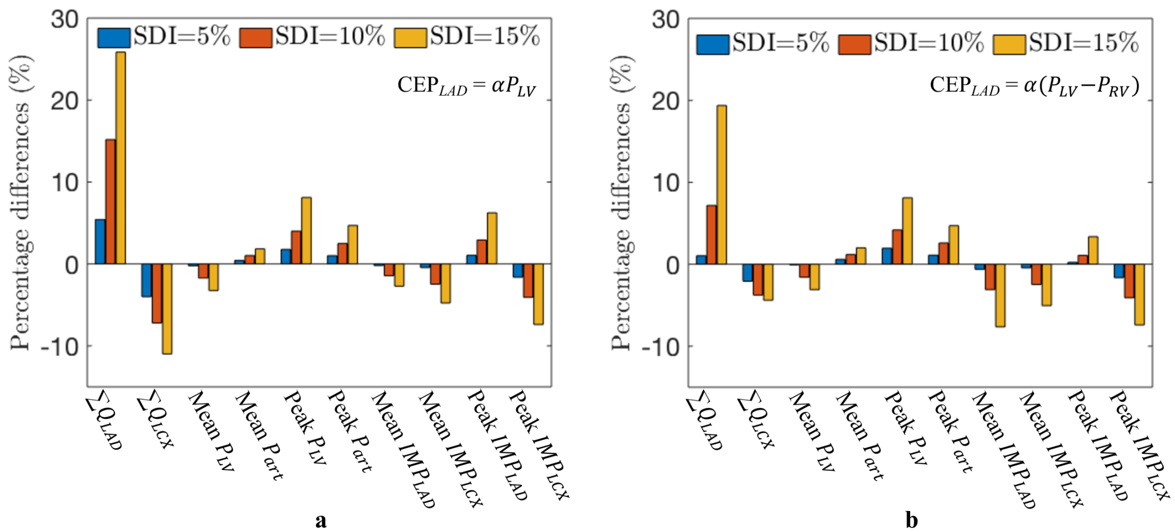


**a** **b**

**Figure E1**: Effects of SDI on total coronary flow, mean arterial and LV pressures, and $IMP_{LAD}$ and $IMP_{LCX}$ over one swine with respect to the control simulation for different SDI. **a.** $CEP_{LAD}$ is based on LV pressure only; **b.** $CEP_{LAD}$ is based on the difference of LV and RV pressures.

**APPENDIX F**: RESULTS FOR THE REST THREE SWINE

In this study, the computational model has been calibrated from three swine. Other than the results for the first swine in Results section, the rest two swine' results including the experimental measurement under RA pacing and model prediction under normal condition are presented in **Appendix F** (**Figure F1-F2**).

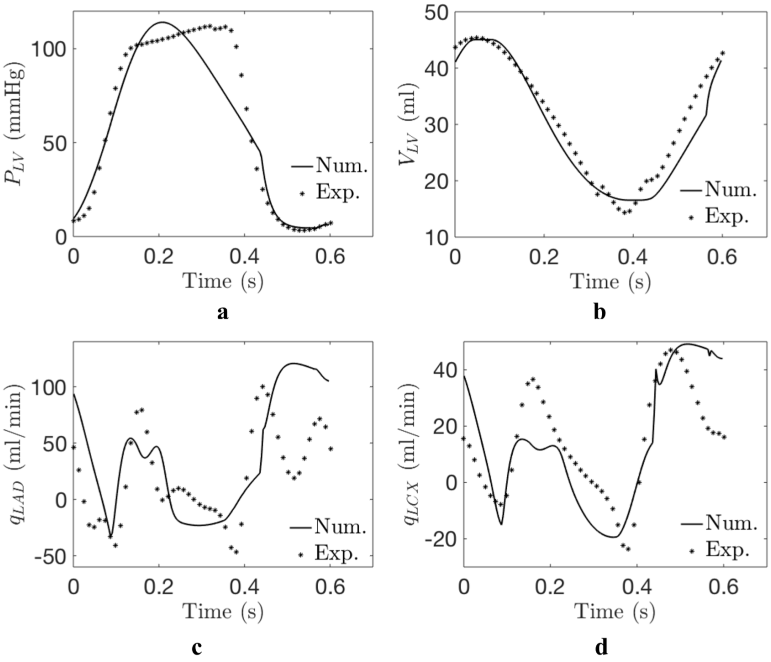


**Figure F1**: The simulation results and experimental data in terms of **a.** LV pressure; **b.** LV volume; **c.** LAD coronary flow rate and **d.** LCX coronary flow rate for the second swine.

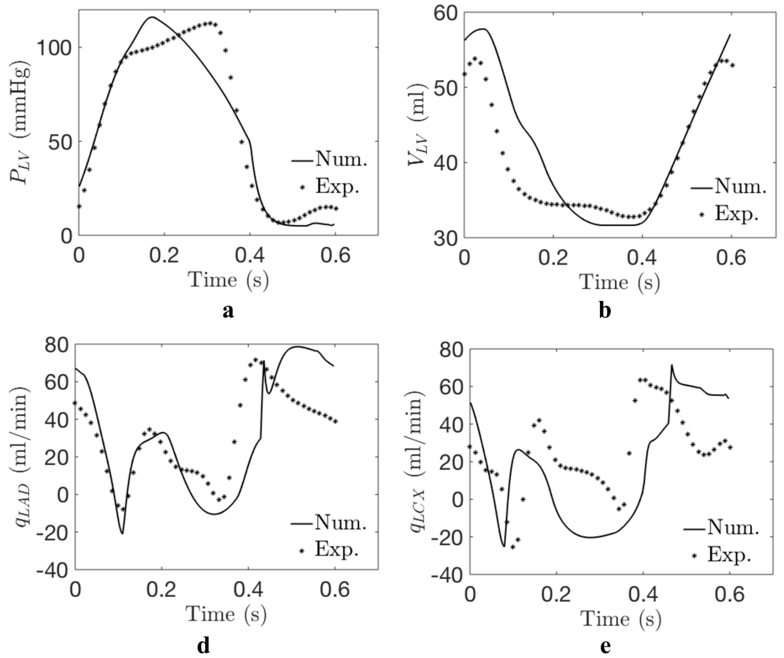


**Figure F2**: The simulation results and experimental data in terms of **a.** LV pressure; **b.** LV volume; **c.** LAD coronary flow rate and **d.** LCX coronary flow rate for the third swine.
